# NPOmix: A machine learning classifier to connect mass spectrometry fragmentation data to biosynthetic gene clusters

**DOI:** 10.1093/pnasnexus/pgac257

**Published:** 2022-11-16

**Authors:** Tiago F Leão, Mingxun Wang, Ricardo da Silva, Alexey Gurevich, Anelize Bauermeister, Paulo Wender P Gomes, Asker Brejnrod, Evgenia Glukhov, Allegra T Aron, Joris J R Louwen, Hyun Woo Kim, Raphael Reher, Marli F Fiore, Justin J J van der Hooft, Lena Gerwick, William H Gerwick, Nuno Bandeira, Pieter C Dorrestein

**Affiliations:** Collaborative Mass Spectrometry Innovation Center, Skaggs School of Pharmacy and Pharmaceutical Sciences, University of California San Diego, La Jolla, CA 92093, USA; Center for Nuclear Energy in Agriculture, University of São Paulo, Piracicaba 13400-970, SP, Brazil; Collaborative Mass Spectrometry Innovation Center, Skaggs School of Pharmacy and Pharmaceutical Sciences, University of California San Diego, La Jolla, CA 92093, USA; Center for Computational Mass Spectrometry, University of California San Diego, La Jolla, CA 92093, USA; NPPNS, Physic and Chemistry Department, School of Pharmaceutical Sciences of Ribeirão Preto, University of São Paulo, Ribeirão Preto 14040-900, Brazil; Center for Algorithmic Biotechnology, St. Petersburg State University, St Petersburg 199004, Russia; Collaborative Mass Spectrometry Innovation Center, Skaggs School of Pharmacy and Pharmaceutical Sciences, University of California San Diego, La Jolla, CA 92093, USA; Collaborative Mass Spectrometry Innovation Center, Skaggs School of Pharmacy and Pharmaceutical Sciences, University of California San Diego, La Jolla, CA 92093, USA; Collaborative Mass Spectrometry Innovation Center, Skaggs School of Pharmacy and Pharmaceutical Sciences, University of California San Diego, La Jolla, CA 92093, USA; Center for Marine Biotechnology and Biomedicine, Scripps Institution of Oceanography, University of California San Diego, La Jolla, CA 92093, USA; Collaborative Mass Spectrometry Innovation Center, Skaggs School of Pharmacy and Pharmaceutical Sciences, University of California San Diego, La Jolla, CA 92093, USA; Department of Chemistry and Biochemistry, University of Denver, Denver, CO 80210, USA; Bioinformatics Group, Wageningen University, 6708 PB Wageningen, The Netherlands; College of Pharmacy and Integrated Research Institute for Drug Development, Dongguk University, Gyeonggi-do 10326, Korea; Institute of Pharmaceutical Biology and Biotechnology, University of Marburg, 35043 Marburg, Germany; Center for Nuclear Energy in Agriculture, University of São Paulo, Piracicaba 13400-970, SP, Brazil; Bioinformatics Group, Wageningen University, 6708 PB Wageningen, The Netherlands; Department of Biochemistry, University of Johannesburg, Auckland Park, Johannesburg 2006, South Africa; Center for Marine Biotechnology and Biomedicine, Scripps Institution of Oceanography, University of California San Diego, La Jolla, CA 92093, USA; Collaborative Mass Spectrometry Innovation Center, Skaggs School of Pharmacy and Pharmaceutical Sciences, University of California San Diego, La Jolla, CA 92093, USA; Center for Marine Biotechnology and Biomedicine, Scripps Institution of Oceanography, University of California San Diego, La Jolla, CA 92093, USA; Collaborative Mass Spectrometry Innovation Center, Skaggs School of Pharmacy and Pharmaceutical Sciences, University of California San Diego, La Jolla, CA 92093, USA; Center for Computational Mass Spectrometry, University of California San Diego, La Jolla, CA 92093, USA; Collaborative Mass Spectrometry Innovation Center, Skaggs School of Pharmacy and Pharmaceutical Sciences, University of California San Diego, La Jolla, CA 92093, USA; Center for Microbiome Innovation, University of California San Diego, La Jolla, CA 92093, USA; Departments of Pharmacology and Pediatrics, University of California San Diego, La Jolla, CA 92093, USA

**Keywords:** genomics, mass spectrometry, machine learning, specialized metabolites, biosynthetic gene clusters

## Abstract

Microbial specialized metabolites are an important source of and inspiration for many pharmaceuticals, biotechnological products and play key roles in ecological processes. Untargeted metabolomics using liquid chromatography coupled with tandem mass spectrometry is an efficient technique to access metabolites from fractions and even environmental crude extracts. Nevertheless, metabolomics is limited in predicting structures or bioactivities for cryptic metabolites. Efficiently linking the biosynthetic potential inferred from (meta)genomics to the specialized metabolome would accelerate drug discovery programs by allowing metabolomics to make use of genetic predictions. Here, we present a *k*-nearest neighbor classifier to systematically connect mass spectrometry fragmentation spectra to their corresponding biosynthetic gene clusters (independent of their chemical class). Our new pattern-based genome mining pipeline links biosynthetic genes to metabolites that they encode for, as detected via mass spectrometry from bacterial cultures or environmental microbiomes. Using paired datasets that include validated genes-mass spectral links from the Paired Omics Data Platform, we demonstrate this approach by automatically linking 18 previously known mass spectra (17 for which the biosynthesis gene clusters can be found at the MIBiG database plus palmyramide A) to their corresponding previously experimentally validated biosynthetic genes (e.g., via nuclear magnetic resonance or genetic engineering). We illustrated a computational example of how to use our Natural Products Mixed Omics (NPOmix) tool for siderophore mining that can be reproduced by the users. We conclude that NPOmix minimizes the need for culturing (it worked well on microbiomes) and facilitates specialized metabolite prioritization based on integrative omics mining.

Significance Statement The pace of natural product discovery has remained relatively constant over the last two decades. At the same time, there is an urgent need to find new therapeutics to fight antibiotic-resistant bacteria, cancer, tropical parasites, pathogenic viruses, and other severe diseases. Here, we introduce a new *k*-nearest neighbor algorithm that can efficiently connect metabolites to their biosynthetic genes. Linking metabolites with the Natural Products Mixed Omics tool allows to make genetic predictions to prioritize relevant products and facilitate their structural elucidation. Our approach can be applied to biosynthetic genes from bacteria (used in this study), fungi, algae, and plants where (meta)genomes are paired with corresponding mass spectometry fragmentation data.

## Introduction

Microbial specialized metabolites are often made by biosynthetic genes that are physically grouped into clusters known as biosynthetic gene clusters (BGCs). Liquid chromatography coupled with tandem mass spectrometry (LC-MS/MS) is an efficient technique to access metabolites from fractions and even environmental samples; however, the metabolomic diversity tends to not represent the full biosynthetic potential of a given microbe (not all BGCs are expressed in certain conditions). The great majority of metabolomics approaches rely on culturing and/or chromatographic isolation for bioactivity assays and structure elucidation. Combining metabolomics analyzes with genome mining would allow making *in-silico* predictions about: (i) the structure, the bioinformatics tool antiSMASH (1) can make accurate structure prediction for some cases, especially for peptides; (ii) the bioactivity, a BGC can be used to predict antibiotic activity via the presence of specific genes or database dereplication; and (iii) the novelty of a given unknown metabolite, via gene networking or beta-diversity. These unknown metabolites can be from microbial cultures or even environmental microbiomes. For example, the random forest classifiers by Walker and Clardy (2) use only the BGC sequences to predict if a BGC will produce an anticancer, antifungal, or antibacterial metabolite (with a maximum accuracy of 80% and each bioactivity required the construction of a classifier to predict if a new entry was bioactive or not). The antimicrobial activity prediction from this tool can be combined with the Antibiotic Resistance Target Seeker (ARTS 2) (3), which annotates antibiotic-resistance genes. Coelichelin was isolated using an *in-silico* structure prediction that indicated the peptide was a siderophore (4). This was confirmed by culturing the producer in an iron-deficient media and isolating the induced (and overexpressed) metabolite. BiG-SLICE (5) was used to predict the connections between more than 1 million BGCs, providing clues to the most diverse/novel BGCs in thousands of samples. These predictions are made using genomics, but metabolomics can access these genomic predictions when metabolites are connected to their BGCs.

However, one of the challenges in the genome mining field is connecting microbial metabolites to their BGCs with confidence. Even the genome of *Streptomyces coelicolor* A3(2), one of the first sequenced microbial genomes, still contains a large number of cryptic BGCs—BGCs without known associated metabolites (6). In 2011, antiSMASH improved the identification and annotation of BGCs based on automated genome mining. Moreover, since 2018, BiG-SCAPE (7) can reliably calculate the similarity between pairs of BGCs, grouping them into gene cluster families (GCFs). Recently, some approaches and tools have been created to connect specialized metabolites (known and cryptic MS/MS spectra) to their biosynthetic gene clusters, such as pattern-based Genome Mining (8, 9), MetaMiner (10), DeepRiPP (11), NRPquest (12), NRPminer (13), GNP (14), and NPLinker (15), recently reviewed by Van der Hooft et al., 2020 (16). Pattern-based Genome Mining is based on the idea that the distribution of a given natural product should be comparable to the distribution of the BGCs responsible for their production. Nerpa (17) and GARLIC (18) can connect structures to BGCs; structures are normally represented in the SMILES (Simplified Molecular-Input Line-Entry System) format, a type of computer-readable annotation language for chemical structures. However, most of these tools are neither high throughput nor efficient, or can only be used for a particular class of BGC (e.g., peptides or BGCs homologous to known BGCs).

These aforementioned tools fall into two main categories: (i) *feature-based approaches* (like GNP, MetaMiner, DeepRiPP, NRPminer, NRPquest, GARLIC, and Nerpa) that use substructure predictions as a link between biosynthetic genes and metabolites (for example, amino acid predictions from biosynthetic adenylation domains or precursor peptides compared to amino acids predicted from known structures or peaks in the MS/MS fragmentation spectra); and (ii) *correlation-based approaches* (like pattern-based Genome Mining and the standardized Metcalf score, e.g., available within NPLinker)(15, 19) that use the distribution of metabolites (via similarity scores that can be obtained based on mass spectral GNPS molecular networking) in the dataset and they compare to the distribution of BGCs (also using similarity scores as calculated by tools like BiG-SCAPE or BiG-SLICE) to create possible BGC-MS/MS links. Of note, NPLinker combines a *correlation-based approach* (via gene and molecular networking) with a *feature-based approach* (Input–Output Kernel Regression method). While our new approach uses unique fingerprints and a *k*-nearest neighbor’s algorithm for connecting metabolites to BGCs, it can be considered a type of pattern-based Genome Mining, which was previously reported by Doroghazi et al. and Duncan et al. (8, 9, 19). Similarly to NPLinker, the addition of substructures as features to our method allows for combining a *correlation-based approach* with a *feature-based approach*.

Another important resource made available recently is the Paired Omics Data Platform (PoDP) (20), which contains paired data (genome and metabolome data of the same samples), as well as validated links between BGCs and MS/MS spectra and annotated structures, which all can be used for the creation and improvement of the aforementioned multi-omics tools and the development of the new approach described in this manuscript. Currently, of over 2,500 paired genomes in the PoDP database, half are metagenomes (mostly feces, human gut, and mouse gut) and the other half are genomes (mostly of bacterial isolates); the 4900 unique metabolomics linked samples (with several genomes having metabolomics data obtained under multiple conditions) were all analyzed via LC-MS/MS, about 90% of the LC-MS/MS runs are from positive mode and the most common extraction solvents used were methanol (39% of the extractions) and ethyl acetate (36% of the extractions). Additionally, most of the BGCs (known and unknown) that we were able to obtain from the PoDP database have biosynthetic components from nonribosomal peptide-synthetases (NRPSs, 31.6% of the BGCs), polyketide synthases (PKSs, 22%) or ribosomally synthesized and post-translationally modified peptides (RiPPs, 19.3%).

It has been challenging to create a systematic tool that can work at the repository scale to connect genotypes (BGCs) with their phenotypes (for example, fragmentation spectra, MS/MS spectra, from untargeted liquid chromatography coupled with mass spectrometry profiles, e.g., LC-MS/MS datasets). This difficulty results from the fact that some metabolites differ in the way that they are biosynthesized and finding a systematic approach relies on identifying common patterns in their biosynthetic pathways. Another challenge is processing the paired data, since it requires combining two types of data that are different in nature (genomics versus metabolomics) and paired datasets are large, being computationally difficult to process. As a result, a large disparity exists between the number of known metabolites from an organism versus the number of BGCs with known metabolite products. For example, the recently designated cyanobacterial genus *Moorena* has already yielded over 200 metabolites, yet only a dozen of validated BGCs are currently deposited for this genus in the expert-annotated Minimum Information about a Biosynthetic Gene cluster (MIBiG) database (21). Connecting metabolites to their biosynthetic genes would also facilitate research concerning the ecological role and functions of the specialized metabolome by studying the regulation of the expression of their biosynthetic gene clusters.

To better address the gap between genomics and metabolomics in drug discovery, we deployed a *K*-Nearest Neighbor (KNN) algorithm that uses similarity BGC fingerprints and analogously similarity MS/MS fingerprints to classify gene cluster family (GCF, a group of similar BGCs) candidates for each MS/MS spectrum. A fingerprint is a group of features measured across the dataset samples for a particular BGC or MS/MS spectrum (for example, the similarity scores between a given BGC and all other BGCs in the dataset, resulting in features that represent each maximum score per genome). We used antiSMASH to annotate all BGCs in this study and BiG-SCAPE to calculate the similarity between annotated BGCs. AntiSMASH annotates BGCs by searching them with profile hidden Markov models of domains from gene/protein sequences known to biosynthesize metabolites and these models are specific to a certain class of BGC. For annotating the similarity between MS/MS spectra, we used the modified cosine score from GNPS (14, 22). We showed that the addition of biosynthetic class and substructure features improves the performance of our Natural Products Mixed Omics tool (NPOmix, available at https://www.tfleao.com/npomix1). We would like to acknowledge that NPOmix and the pattern-based Genome Mining approaches are limited to organisms that have BGCs (not all organisms have BGCs as well-defined as bacteria and fungi, for example, higher plants and animals typically have less well-structured clusters of biosynthetic genes) and to BGCs that are somewhat similar to at least one of the reference BGCs in the PoDP datasets.

Of note, in this study, six of the 11 BGC-metabolite links used for validation (not counting analogs) were previously fully characterized via knockouts, heterologous expression, and/or isolation, and unambiguous NMR structure elucidation as recorded at the PoDP database. The number of genomes, metabolomes, BGCs, GCFs, GNPS metabolites, metabolites with MIBiG BGC, and BGC-metabolite links (with and without filtering) are listed in Table S1. The major limitation of the evaluation of our method was the lack of available test data for structures that are linked to their MS/MS spectra and biosynthetic gene clusters—a bottleneck that the application of NPOmix can help solve and we discuss in more detail in the “Supplementary Material and Background” section. We believe that NPOmix will assist with the discovery of novel metabolites as well as known metabolites with new biosynthesis (more details in the “Supplementary Material and Background” section). We exemplify a computational method combining NPOmix and MassQL (23) for prioritizing siderophores from thousands of metabolome profiles and this method can be reproduced by the users with their own samples.

## Results

### Our proposed solution: the NPOmix approach

To enable *in-silico* predictions of molecular novelty, bioactivity, and chemical structure, metabolites need to be connected to their corresponding BGCs, which is, as aforementioned, a major challenge in the metabolite discovery field. Therefore, we present a KNN classifier named NPOmix that can use just DNA sequences and unlabeled mass fragmentation profiles to accurately create links between metabolites and their corresponding biosynthetic gene cluster, helping to prioritize metabolites by also making use of *in-silico* genomic predictions.

**Fig. 1.**
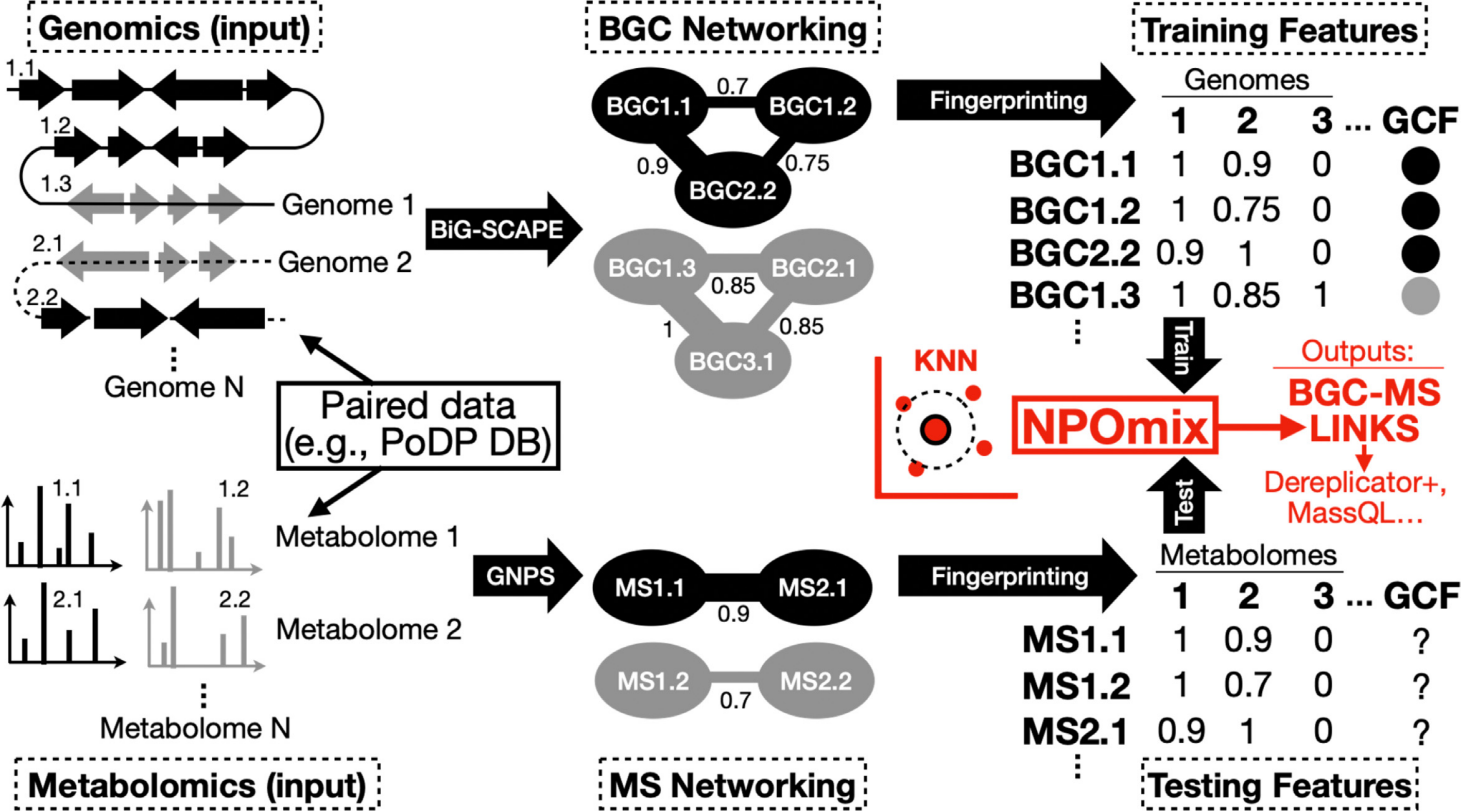
Schematic of how NPOmix works including inputs, transformations, processing tools, outputs, and the idea of combining the outputs with other tools. BGC = biosynthetic gene cluster; MS = mass fragmentation spectrum; KNN = *k*-nearest neighbor; BiG-SCAPE = software to calculate pairwise BGC–BGC similarity; GCF = gene cluster family (group of similar BGCs); GNPS = tool to calculate the modified cosine score indicating the pairwise spectrum–spectrum similarity; BGC-MS links = links between metabolites and BGCs produced by the KNN classifier; Dereplicator+, MassQL = other downstream analyses tools that can be combined with NPOmix to validate outputs or improve NPOmix predictions.

To use the NPOmix approach (schematic in Fig. 1 and more details in Fig. S1), it is required to have a paired dataset with genomic and MS/MS information as inputs. Fig. 1 shows a conceptual example using only a few samples and only using similarity as a feature (same for Fig. S1). The genomic information can be either that of a genome or metagenome and the MS/MS spectra should be obtained via untargeted LC-MS/MS. Paired datasets have become available at the PoDP database (20), one of the first initiatives to gather paired genomic and MS/MS information. Our analysis contained 3,331 BGCs (used for training) that were present in 1,040 networked genomes/metagenomes with paired LC-MS/MS data that could be downloaded from the PoDP database.

We first grouped known and cryptic BGCs in GCFs based on domain similarity using BiG-SCAPE (7) to obtain labels that can be used for supervised learning (BGC networking section in Fig. 1 or Fig. S1A). Regarding this training set, out of the 3,331 BGCs annotated by antiSMASH in the (meta)genomes from the PoDP database and networked by BiG-SCAPE, 260 BGCs (7.8%) were dereplicated by similarity to the MIBiG database (in other words, BGCs from families containing at least one known MIBiG BGC). To create a BGC fingerprint (training features section in Fig. 1 or Fig. S1C), we identified the similarity between the individual BGC and each of the BGCs in each genome in the training dataset (similarity scores are computed as “1 minus the raw distance”). The BGC fingerprint that emerges is a series of columns for each compared genome, the column value of which represents the similarity score between the individual BGC and the BGC to which it is maximally similar in each genome (column). Similarity scores range from 0.0 to 1.0; identical BGCs have perfect similarity and are scored as 1.0 whereas a score of 0.8 would indicate that a homologous BGC is present in the genome. A score below the (user-defined) similarity cutoff of 0.7 indicates that the queried BGC is likely absent in the genome. We selected the cutoff as 0.7 because it is the same cutoff used for BiG-SCAPE (7) similarity (a value that was determined using a cutoff calibration with the MIBiG database) (24). An analogous process is used to create MS/MS fingerprints (testing features section in Fig. 1 or Fig. S1B and S1D); an individual MS/MS spectrum is compared to all of the MS/MS spectra in the testing set using the GNPS modified cosine score (14, 22) and the maximum similarity cosine score between the individual MS/MS spectrum and the spectra in a given metabolome is incorporated as part of the MS/MS fingerprint. Furthermore, we add the presence/absence of biosynthetic classes and substructures to the BGC and MS/MS fingerprints (for more details, refer to the “Methods” section). We then use the BGC fingerprint in the training together with the generated family labels. In more detail, the BGC fingerprints form a training matrix (training features section in Fig. 1 or Fig. S1E, where each column is a genome and each value is the maximum similarity between the individual BGC and the BGCs in this given genome) and, in this case, the matrix contains 1,040 columns due to the 1,040 sets of paired experimental samples plus the columns corresponding to the biosynthetic class and annotated substructures.

The trained KNN classifier takes as input the same kind of features (MS/MS fingerprint, Fig. S1D, and the testing matrix is exemplified in Fig. S1F or testing features section in Fig. 1), where the similarity is now represented by the modified cosine score but these features are annotated from individual mass fragmentation profiles (known or cryptic metabolites). The aim of NPOmix is then to label these MS/MS fingerprints with the proper gene cluster family, thus accurately linking fragmented metabolites to their corresponding BGCs. The testing MS/MS spectrum used as a query could be either a reference spectrum from the Global Natural Products Social Molecular Networking database (GNPS) (14, 22) or a cryptic MS/MS spectrum from a new sample that contains a sequenced genome and experimental MS/MS spectra. In more detail, the KNN algorithm links BGCs to fragmented metabolites (represented by their MS/MS spectra) by plotting the BGC fingerprints in the KNN feature space (NPOmix step in Fig. 1 or Fig. S1G). The KNN feature space is exemplified by only two dimensions since a 1,040-dimensional space is not feasible to comprehensively visualize (one dimension per sample). We would like to emphasize that the actual KNN prediction is made in many dimensions (equivalent to the number of features), and we only depicted these multidimensional fingerprints in 2D in Fig. S1G (as a schematic, we did not perform dimensionality reduction). More details of how this multidimensional plotting occurs are illustrated in Fig. S2, where the samples correspond to the 3D axes and the similarity scores are the coordinates. Each testing MS/MS fingerprint (a row in the testing metabolomic matrix and columns are the experimental MS/MS spectra per sample) is plotted into the same KNN feature space (NPOmix step in Fig. 1 or Fig. S1G) so the algorithm can obtain the GCF labels for the nearest neighbors to the testing MS/MS fingerprint (e.g., for three most similar BGC neighbors, *k* = 3). We note that GCF labels can be present more than once in the returned list if two or more BGC nearest neighbors belong to the same GCF. Given that many related BGCs are part of the same GCF, this repetition of the GCF classification is a common behavior of our KNN approach. The BGC-MS/MS links generated as outputs by NPOmix can be combined with the outputs of other tools like Dereplicator+, MassQL, and so on; as exemplified (later in this manuscript) for the case of brasilicardin A and new putative siderophores.

In principle, our approach is suitable for bacterial (exemplified here), fungal, algal, and plant genomes, and MS/MS spectra obtained from the same organism (if these organisms contain BGCs). Metagenomes and metagenome-assembled genomes (MAGs) can also be used instead of genomes; however, complete genomes are preferred in the training set primarily due to the expected data quality. This KNN approach also supports LC-MS/MS from fractions or different culture conditions; multiple LC-MS/MS files for the same genome were merged into a single set of experimental MS/MS spectra.

### Validation and multi-omics dereplication: linking known metabolites to known BGCs, validated links from the PoDP database, and BGCs from the MIBiG database

To validate NPOmix, we used 36 out of 71 meta-datasets from the PoDP database (from February 2021, meta-datasets listed at Dataset S1, sheet one). We were not able to download all meta-datasets due to data issues (such as lack of access to some genomes and incorrect links/IDs) that we believe will be fixed in the future. There are currently 75 datasets in the PoDP database. We selected genomic samples that contained a valid Genome ID or BioSample ID to aid their downloading from the National Center for Biotechnology Information database and totaling 732 genomes/MAGs obtained from these 36 meta-datasets. In addition to these genomes/MAGs, the 36 meta-datasets also contained paired data for metagenomes. Hence, we selected and assembled 1,034 metagenomes and these metagenomes were concentrated on two major meta-datasets: (1) MSV000082969 and PoDP ID cd327ceb-f92b-4cd3-a545-39d29c602b6b.1–556 cheetah fecal samples and environmental samples (25); and (2) MSV000080179 and PoDP ID 50f9540c-9c9c-44e6-956c-87eabc960d7b.3–The American Gut Project (26) that contains fecal samples from 481 human subjects. These (meta)genomes were downloaded with the code shared at the GitHub repository https://github.com/tiagolbiotech/NPOmix, notebook 1. The LC-MS/MS files can be downloaded using “FTP” from links recorded in Dataset 1, sheet two. We were able to network 1,040 (meta)genomes that contained 3,331 BGCs (including 260 BGCs from the MIBiG database) distributed into 997 GCFs. In the untargeted metabolomics data, we matched 3,248 LC-MS/MS files to 22 GNPS (14, 22) reference library MS/MS spectra and one spectrum not available at GNPS (brasilicardin A, obtained from the microbial metabolome) to create the MS/MS fingerprints for testing the KNN classification (one fingerprint per spectrum). We envision creating a more balanced, diverse, and less sparse training dataset in ongoing efforts. To maximize precision rates in the future, we plan to purchase cultures from collections that have well-assembled genomes so we can obtain the paired LC-MS/MS. However, the current dataset produced highly supportive results by testing validated links from the PoDP and semimanually connected links used in the NPLinker publication, all of which will be reused for posterior benchmarking of BGC-MS/MS linking algorithms. We attempted to test all 242 BGC-metabolite links used as training data for the Rosetta scoring method in NPLinker (totaling 2,069 unique MS/MS spectra, Dataset S1, sheet two) plus 109 manually added MS/MS links (connected to BGCs, annotated by experts at the PoDP database, Dataset S1, sheet three). However, most of these validated MS/MS spectra were not present in the 1,040 paired (meta)genomes-MS/MS samples from the PoDP, or their BGC scores did not co-occur with their MS/MS scores because they were not present in the nearly exact same samples. To further illustrate this, 178 MIBiG BGCs networked with PoDP BGCs, and 50 GNPS-annotated metabolites were reported at PoDP. The intersection of these two resulted in 22 BGC-metabolite links including analogs. We obtained the largest validation dataset of metabolites linked to BGCs possible (that also contained genomes and metabolomes). We note that we used all links available in the PoDP database that could be annotated in the downloaded paired data, thereby not on purpose biasing this validation set toward metabolites that would be better classified using NPOmix, but we acknowledge that we are dependent on the availability of validated BGC-MS/MS links. You can see these and other metrics in Table S1.

Hence, our validation dataset was limited to 11 validated links (22 with analogs) from the MIBiG and PoDP databases and found in the paired (meta)genomes-MS/MS samples (barbamides, antimycin, pyocyanines, 2,4-diacetylphloroglucinol, brasilicardin A, orfamides, albicidins, bafilomycin B1, nevaltophin D, jamaicamides, and cryptomaldamide, totaling 22 references MS/MS spectra that were present in the GNPS database). We only included analogs for which the MIBiG BGC was present in our paired samples downloaded from the PoDP; hence, we included orfamide A to C but not orfamide E, F, and so on (because they are not reported as products from the MIBiG orfamide BGCs). We stress that a larger training dataset with more complete genomes is likely to increase the size of the validation set by adding more valid BGCs into the analysis. We were also able to combine NPOmix with *in-silico* metabolomics tools like Dereplicator+ (21) to make new links between MS/MS spectra, BGCs, and molecular structures, as we will exemplify by brasilicardin A. This was accomplished by annotating cryptic MS/MS spectra (without a GNPS library hit and therefore not present in the GNPS database) to known BGCs (found in the MIBiG database). Such new links could be confirmed experimentally to improve the size of the validation set, as well as to expand MS/MS databases by adding these cryptic spectra to them.

**Fig. 2.**
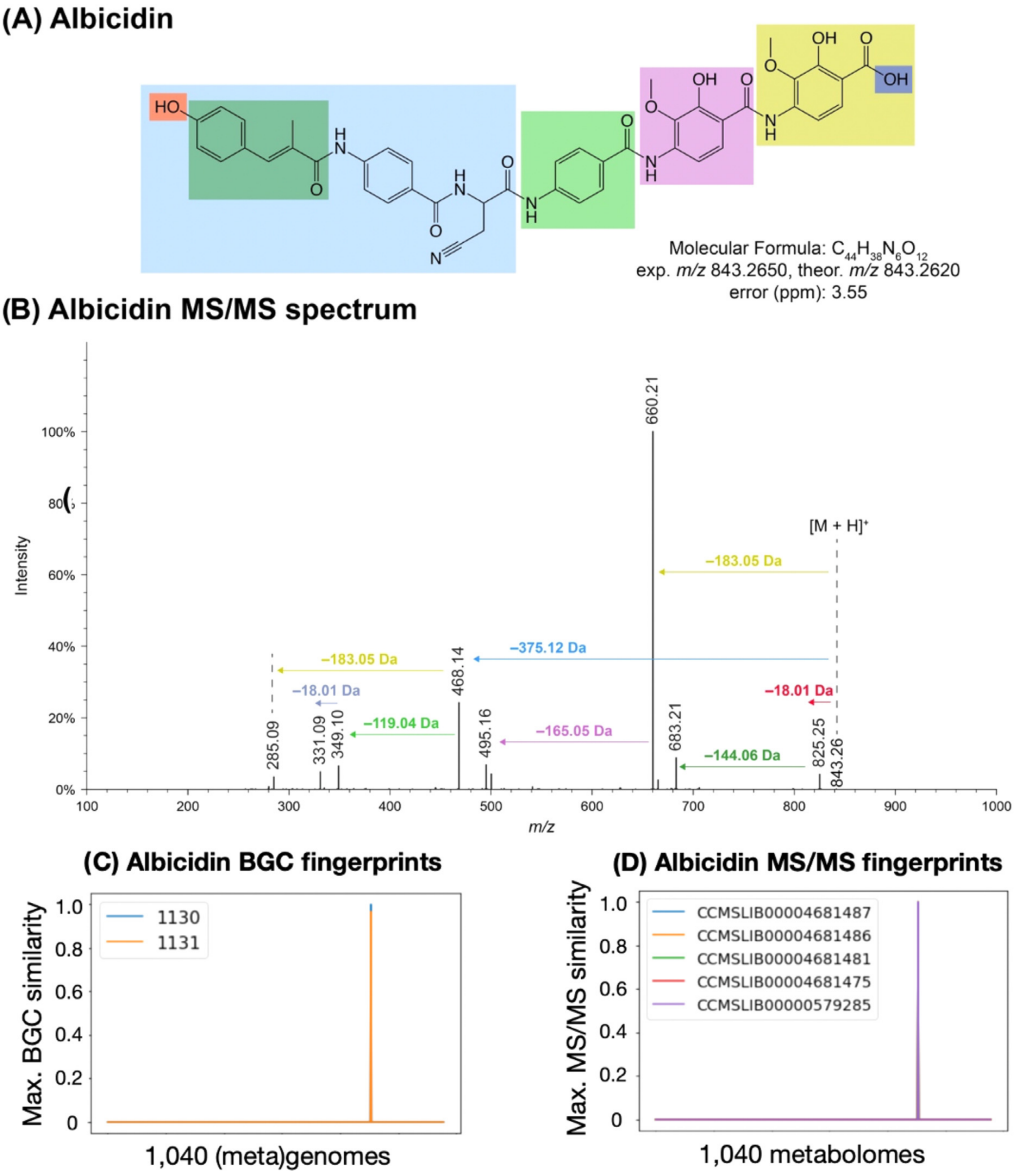
Multi-omics enabled the dereplication of albicidin by automatically predicting a true BGC-metabolite link. Structure of the dereplicated metabolite with highlighted observed fragments (A) and its corresponding representative MS/MS spectrum (B, spectrum example from GNPS ID CCMSLIB00000579285, *m/z* of 843.2620, and ppm error of 3.55), obtained via Metabolite Spectrum Resolver (27). The two BGC fingerprints (1,130 and 1,131) are represented in a 2D plot (C) and they match the 2D plot for the five MS/MS fingerprints obtained from GNPS for albicidin and its analogs (D). *m/z* = mass over charge calculated via mass spectrometry; exp. *m/z* = experimental *m/z*; theor. *m/z* = theoretical *m/z*.

A 2D comparison of both types of fingerprints (BGC and MS/MS) can be a proxy for distinguishing some true positives from false positives. As observed in Fig. S3, we can visualize a mismatch between the BGC fingerprints (one GCF) and the MS/MS fingerprint in the “reduced” KNN-space (represented schematically in only two dimensions, we did not perform actual dimensionality reduction), indicative of a possible false positive link. This GCF is dereplicated as the known metabolite, pyocyanin, and it was incorrectly associated with the metabolite 2,4-diacetylphloroglucinol, confirming the false positive (at *k* = 3). In contrast, Fig. 2 illustrates that five metabolites, albicidin (structure in Fig. 2A and MS/MS spectra in Fig. 2B) and four albicidin analogs, could be correctly assigned to their corresponding GCF that contains two BGCs (one from the MIBiG database and another from *Xanthomonas albilineans* GPE PC73, GenBank ID GCA_000,087,965.1). In this case, the BGC fingerprints match the MS/MS fingerprints (Fig. 2C and D). This observation of “co-occurrence” between the strains in the BGC fingerprint and MS/MS fingerprints can be quantified by the Jaccard index (a score ranging from zero to one and calculated by the intersection of the presence/absence between the strains in fingerprints over the total number of strains). The Jaccard index is a measurement between the number of features (similarities, biosynthetic classes, and substructure predictions) that are present or absent while comparing two fingerprints (one BGC and one MS/MS fingerprint). In other words, the Jaccard index is a proxy of how good an NPOmix link is between a given metabolite and the connected BGC and it can be interpreted as a confidence score or used as a filter on the results.

Using the PoDP dataset, a Jaccard index cutoff of 0.7, and similarity plus biosynthetic class as features for the *k*-nearest neighbor model, we obtained a precision of 92.9% as 13 out of 14 reference MS/MS spectra were correctly labeled when top-*n* = 3 (*k* equal to 3, metabolites and their predicted GCFs listed in Table 1). Precision is the ratio between the true positive links over all the tested positives (true positives plus false positives). Top-*n* represents how often the correct GCF label was found among the top-*n* labels classified by the KNN approach. We have determined that the use of three neighbors is the optimal performance (also using the co-occurrence threshold), providing a good balance between precision and the number of links to validate (very high precision and randomness equal to 0, as detailed in Table S2). Randomness is observed by shuffling the testing columns, experimental MS/MS names, and counting how many correct links are present between the top-*n* GCF candidates. Lastly, we regard our NPOmix approach as multi-omics enabled dereplication because the 13 MS/MS spectra were automatically assigned to a known GCF that confirmed their metabolite labels, thereby minimizing the need for purchasing standards, performing isolation and NMR characterization, gene knockout, or heterologous expression. This kind of multi-omics analysis allows us to dereplicate more metabolites than genomics or metabolomics separately and it helps researchers to focus on novel structures that can also present novel bioactivities.

**Table 1.** Fourteen links between GNPS MS/MS spectra (with metabolite ID) and networked gene cluster family (true GCF that contained a known MIBiG BGC and passed the co-occurrence threshold).

**Metabolite ID**	**True GCF**	**Predicted GCFs for *k* = 3**	**Annotation**
CCMSLIB00004679298	GCF450	GCF360, **GCF450**, GCF360	Orfamide A*
CCMSLIB00004679299	GCF450	GCF360, **GCF450**, GCF360	Orfamide B*
CCMSLIB00004679300	GCF450	GCF360, **GCF450**, GCF360	Orfamide C*
CCMSLIB00000001573	GCF468	**GCF468**, GCF468, GCF235	Barbamide
CCMSLIB00000001575	GCF468	**GCF468**, GCF468, GCF235	Barbamide
CCMSLIB00000001706	GCF471	**GCF471**, GCF498, GCF550	Jamaicamide A
CCMSLIB00000001708	GCF471	**GCF471**, GCF498, GCF550	Jamaicamide C
CCMSLIB00000579285	GCF476	**GCF476**, GCF219, GCF235	Albicidin
CCMSLIB00004681475	GCF476	**GCF476**, GCF219, GCF235	Propionyl-albicidin
CCMSLIB00004681481	GCF476	**GCF476**, GCF219, GCF235	Beta-methoxy-albicidin
CCMSLIB00004681486	GCF476	**GCF476**, GCF219, GCF235	Carbamoyl-ß-methoxy-albicidin
CCMSLIB00004681487	GCF476	**GCF476**, GCF219, GCF235	Carbamoyl-ß-methoxy-asn-albicidin
CCMSLIB00000840594	GCF488	GCF740, GCF740, GCF739	Nevaltophin D
CCMSLIB00005724004	GCF498	GCF471, **GCF498**, GCF550	Cryptomaldamide

The table also includes their KNN predictions (*k* = 3); the predicted GCFs are ordered according to the value for *k*, from 1 (nearest) to 3 (furthest), and the first correct family is marked in bold font. GCF labels can be repeated because multiple BGCs from the same GCF can be predicted as the nearest neighbors. Classification is considered correct if the true GCF is among the top-3 candidates. Annotations are according to each MIBiG BGC(s) found in the true GCFs. GNPS = Global Natural Products Social Molecular Networking database; KNN =   *K*-nearest neighbor; MS/MS spectra = fragmentation spectra; BGC = biosynthetic gene cluster; GCF = gene cluster family; MIBiG = Minimum Information about a Biosynthetic Gene cluster database; * = new analogous MS/MS spectra that were automatically connected to validated BGCs.

We expect that our approach will improve with a larger training set and with further improvement of the features in the BGC and MS/MS fingerprints (e.g., by adding BGC regulation and predicted bioactivity). We confirmed that all 13 correct GCF predictions reported here were found in the original producer of the identified metabolites; they matched the reported masses and most of these BGC-metabolite links were reported at the PoDP database as validated via knockouts, heterologous expression, and/or isolation, and NMR structure elucidation. NPLinker (15), another recently published paired-omics tool, also uses validated links from the PoDP to assess linking precision scores. With 50 known GCF-MS/MS links that were present in the 1,040 samples with paired data (some metabolites have multiple MS/MS spectra), the annotation rate was reasonably good (around 28%, 14 out of 50 links were retained after the Jaccard co-occurrence threshold, a threshold to keep only the metabolites that are found among the same samples that contain the candidate BGCs). Table S1 provides an overview of the various numbers of genomes, metabolomes, BGCs, metabolites, etc.

### Dereplicating a new analogous MS/MS spectrum (with a library hit from GNPS but not found at the PoDP database) to a known BGC

In addition to dereplicating known metabolites (from the GNPS and/or the PoDP database), NPOmix can also dereplicate new putative analogs (orfamides marked by asterisks in Table 1). For example, the BGC for the metabolite orfamide C (genes 1 to 6 in Fig. S4, MIBiG ID BGC0000399) was automatically connected by our KNN approach (both using only similarity as a training/testing feature or by adding the biosynthetic class) to a GNPS metabolite labeled “putative orfamide C” (CCMSLIB00004679300). This MS/MS spectrum was also found in the same strain where the BGC was first identified (*Pseudomonas protegens* Pf-5, Genbank ID GCA_000,012,265) (28). The nine amino acid (AA) predictions for this BGC, based on the specificity of adenylation domains, match the structure of orfamide C in the correct order: leu, asp, thr, ile, leu, ser, leu, leu, and ser. AntiSMASH was not able to predict the tenth and last AA in the biosynthetic series, although, the mass difference between the partial structure and the experimentally observed *m/z* pointed that the last AA was indeed valine. Of note, NPOmix does not make structure predictions and the orfamide C structure was predicted semimanually (we relied on antiSMASH to make most of the genome-based predictions, whereas we predicted the last amino acid using a mass difference between the antiSMASH structure and the observed ion via mass spectrometry). The matching between the predicted structures (via dereplication versus de novo prediction using the NPOmix link) confirmed the multi-omics enabled dereplication of this “putative orfamide C” (using *k* = 3, BGC predictions and predicted metabolite structure are represented in Fig. S4), annotating this metabolite without the need for isolation. We would like to stress that this version of the KNN GCF predictions did not use structures/substructures for linking MS/MS spectra to BGCs; hence, as demonstrated in Fig. S4, substructure predictions can be an extra dimension for selecting links that are true positives over false positives.

### Connecting a cryptic/new MS/MS spectrum (not present in the GNPS database) to a known BGC (validated link from the PoDP database)

We used a combination of MS/MS fingerprints (notebook 2 from https://github.com/tiagolbiotech/NPOmix), BGC fingerprints (notebook 3), Mzmine (29), and Dereplicator+ (30) to link and dereplicate brasilicardin A. After selecting 300 MS/MS spectra from the 16 most diverse genomes in the dataset (diversity estimated using GCF presence/absence) of 1,040 samples, Dereplicator+ had three *in-silico* metabolomics predictions and one of them was the unique tricyclic glycosylated terpene brasilicardin A. The observed *m/z* matches the value previously reported in the literature (31), identifying an MS/MS spectrum that is currently absent from the GNPS database. NPOmix connected the MS/MS spectrum (predicted to be brasilicardin A by Dereplicator+; please note that this information was not used during the NPOmix training) with the correct BGC (brasilicardin A MIBiG ID BGC0000632 from the strain *Nocardia terpenica* IFM 0406, GenBank ID GCA_001,625,105) (32), highlighting how NPOmix can connect cryptic metabolites without library matches (absent from MS/MS databases like GNPS) to their corresponding BGCs. Predicted fragmentation (Fig. S5) strongly suggested that the query MS/MS spectrum is corresponding to the planar structure of brasilicardin A (all deltas between fragmented exact *m/z* and observed *m/z* were extremely low, below 0.001, more information in Dataset S1, sheet four). Thus, NPOmix can provide links between cryptic MS/MS spectra and known/cryptic BGCs from the most diverse strains and potentially new BGCs that can be explored experimentally (e.g., BGC knock-out, heterologous expression, or isolation, and NMR structure elucidation), especially if coupled to SMART-NMR analysis (33) to confirm their novelty. We would like to emphasize that NPOmix used the similarity scores (in other words, the distribution of the metabolite among the training samples) to connect the so far cryptic MS/MS spectrum (brasilicardin A) to its correct BGC. The biosynthetic class, substructure predictions, annotation of the spectrum, and comparison to existing spectra libraries were not used as inputs for NPOmix to make this correct link. We did use this information to validate the link: for example, the MIBiG annotation matched the Dereplicator+ annotation of brasilicardin A. Since we initially selected the PoDP metabolites that were dereplicated by GNPS to be part of the validation set, we overlooked brasilicardin A (which was absent in the GNPS database). After this analysis combining NPOmix, MZmine, and Dereplicator+ that correctly linked the metabolite brasilicardin A to its BGC, we returned to the PoDP database and found an entry corresponding to this link that was already validated (via “knockouts, heterologous expression, or other gene cluster manipulation,” information at the dataset 1b0dccac-5212–4dfd-a9f2-6fa953ab16bd.5). This entry had the same *m/z*, LC-MS/MS metabolome, and retention time of brasilicardin A that we observed for the previously cryptic metabolite.

### Comparing the three different kinds of features for linking metabolites to BGCs using NPOmix

**Fig. 3.**
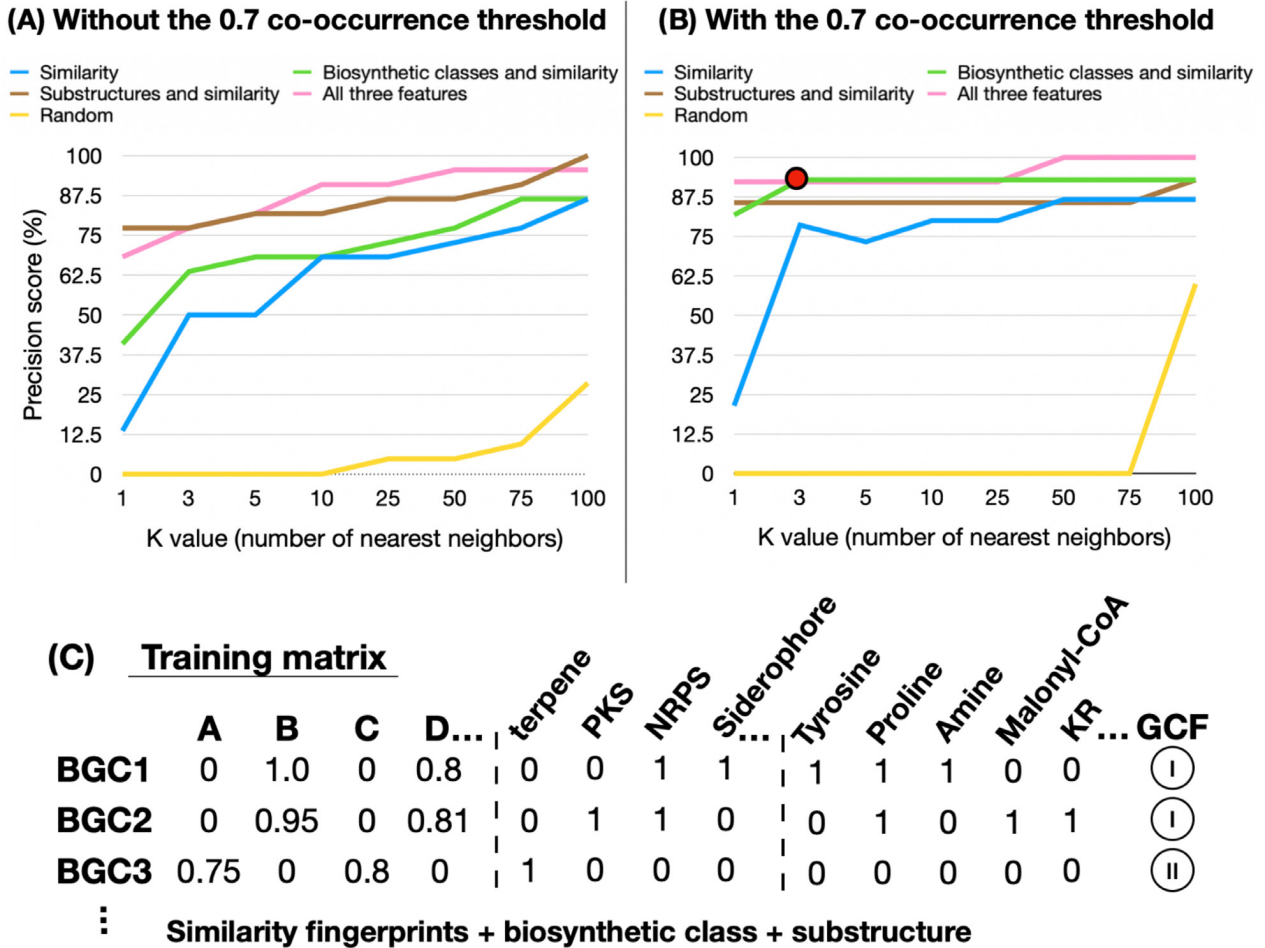
(A) Precision curves without the Jaccard co-occurrence threshold for different features: similarity only (blue), biosynthetic class and similarity (green), substructure predictions and similarity (brown), and using both biosynthetic class, substructure predictions, and similarity (pink). Precision is the ratio between the true positive links over all the tested positives (true positives plus false positives). (B) Precision curves for the same cases using the co-occurrence threshold. Random (yellow) was calculated by using similarity only and shuffling the testing columns. The red dot indicates the best precision of 92.9% (using biosynthetic class and similarity as features, co-occurrence threshold, and *k* = 3). *K* represents the number of nearest neighbors. (C) We illustrate the modifications made in the training matrix with the addition of biosynthetic class and substructure prediction. For this work, we used 1,040 samples, therefore, 1,040 similarity columns plus 12 biosynthetic classes and 82 predicted substructures.

In order to increase the precision of our NPOmix algorithm, we added to the BGC and MS/MS similarity fingerprints the presence/absence of biosynthetic classes (polyketide synthases, nonribosomal peptide-synthetases, terpenes, siderophores, RiPPs, phosphonates, oligosaccharides, phenolic metabolites, others/unknowns, other minor classes, and combinations of more than one class) and substructures (tyrosine, proline, amine, malonyl-CoA, and so on—all 85 predicted substructures can be found at Dataset S1, sheet five). A comparison of the various models is provided in Fig. 3, with Fig. 3A without using the co-occurrence threshold, and Fig. 3B with use of the co-occurrence threshold. Of note, since all testing BGC-MS/MS links (in the validation set) are present in the MIBiG database, we used the information from this database to infer the biosynthetic class and substructure predictions in this particular analysis. However, the biosynthetic class can be predicted for unknown metabolites using MolNetEnhancer (34) and/or CANOPUS (35) and the substructure predictions for unknown metabolites can be obtained using tools like MS2LDA (36), MassQL (23), and/or CSI: FingerID/SIRIUS 4 (37). To obtain genomics-inferred substructure features for large paired omics datasets in the future, we envision that the recently proposed iPRESTO approach (38) could be applied to discover commonly occurring patterns of biosynthesis genes that together likely produce a substructure of a specialized metabolite.

The comparison of the different features for running NPOmix is exhibited in Fig. 3, using the same set of samples previously described (1,040 paired samples and 22 validated BGC-metabolite links). As observed in the figure, the Jaccard co-occurrence threshold (a threshold to ensure that the query metabolite is connected to BGCs in the genome of the same microbial producer) substantially improved the precision scores, but it dropped the number of validated links from 22 to around 14 (these links are listed in Table 1). However, we obtained high precision scores even without threshold (Fig. 3A), for example, 81.8% for *k* = 5 using the three kinds of features (similarity, biosynthetic class, and substructure prediction) or just similarity and substructure features. Interestingly, we observed a slightly higher precision for *k* = 1 when only the substructures and similarity features are used in opposed to using all three kinds of features (without threshold); this is because the biosynthetic class for pyocyanines at MIBiG (used for manual annotation of the MS/MS spectrum) are annotated as “other” but antiSMASH automatically annotates its corresponding BGCs as “minor” and in some cases as both “minor” and “NRPS,” creating a mismatch between the biosynthetic classes. Additionally, we summarized KS domains and PKS substrates in only two columns (one for each) but we obtained the same precision scores. We elected the best precision score as 92.9% (red dot in Fig. 3B, score with co-occurrence threshold) because this very high value was obtained using only the similarity and the biosynthetic class features and three nearest neighbors, a good number of candidates for genome mining. Moreover, the use of just the similarity and biosynthetic class is very appealing because these kinds of features can be well predicted for metabolites with known or unknown metabolites, for example, by using GNPS for similarity and CANOPUS and/or MolNetEnhancer for biosynthetic class predictions, as demonstrated by NPClassScore (39). It is much more challenging to predict substructures for unknown/cryptic metabolites, which is a topic of ongoing research by our group and others.

In Fig. 3C, if a given BGC is a hybrid polyketide synthase (PKS) and nonribosomal peptide-synthetase (NRPS), it was annotated as 1 in the PKS and NRPS columns, and with a 0 in the remaining classes (additional columns). Analogously to the biosynthetic classes, each substructure prediction represented a new column in the BGC fingerprint or MS/MS fingerprints, and the columns were filled with 1 (if the substructure was present) and 0 (if the substructure was absent). For the MS/MS fingerprints in the testing set, we manually annotated these biosynthetic class features based on the known structures and MIBiG information. In cases where the structure is unknown, tools like CANOPUS (35) and MolNetEnhancer (34) can provide a similar biosynthetic class prediction relying on NPClassifier (40). For the substructure features in the MS/MS testing set, we made use of genomics-inferred information provided by antiSMASH to annotate substructures for a BGC from the same GCF as the validated MIBiG BGC. In cases where the BGC is unknown, these predictions can be obtained: manually (by checking the known structure for common substructures); via unsupervised tools like MS2LDA (36,41); or via supervised tools like MassQL (based on specific MS/MS fragments found in the spectra) (23) or CSI: FingerID/SIRIUS 4 (37). For example, we manually annotated the substructures predicted from the palmyramide A structure and we were able to correctly link the validated MS/MS spectrum with the corresponding palmyramide A BGC. This BGC is not yet published at MIBiG, but the biosynthetic gene cluster was previously annotated and reported in *Moorena producens* PAL (42), and in this study, the same genome, LC-MS/MS metabolome, and MS/MS spectral data were used to obtain this BGC-metabolite correlation. Lastly, we observed the distribution (Fig. S6) and the variance of the training dataframe (including all three kinds of features) used for this comparative analysis. The average variance of the columns was the same as rows (about 0.008), the maximum variance for the columns was 0.213, and for the rows was 0.068. The distribution (Fig. S6) is very sparse (many zeros) and it also explains why the average variance is relatively small since all nonzero scores ranged from 0.7 to 1.0. Additionally, the latter score was the most common score in the training dataframe since every self-hit had a perfect score of 1.0 and the biosynthetic classes/substructure predictions were binary (either 0 or 1.0).

### Comparing the performance of NPOmix with other published tools

We used the same previously described dataset of 1,040 paired samples that includes 22 validated BGCs-metabolites links to compare precision scores (same links used in the calculation of the precision scores in Fig. 3) to other eight multi-omics (tools and their websites are listed in Table S3). Precision is the ratio between the true positive links over all the tested positives (true positives plus false positives), and this metric was calculated using only the best candidate (top-1) from the tools’ outputs. For the tools that do not have a training step, we selected from the 1,040 paired samples only the ones that contained at least one of the 22 BGCs-metabolites validated links, a total of nine paired samples. We compared NPOmix to NRPminer (13) and NPLinker (15), the other currently available tools that create links between MS/MS data and BGCs. NPLinker includes the correlation scores first developed in the pattern-based Genome Mining publications (8, 9) and derivatives thereof. GNP (14) and NRPquest (12) seem to be discontinued (see Table S3 for further details).

MetaMiner (10) and DeepRiPP (11) could not be used in the comparison because, unfortunately, there are no RiPPs among the 22 links in our validation set. As far as we can tell, there are only two validated RiPPs in the entire PoDP database: capistruin C and polytheonamide A–B. However, neither of the two were available for our validation test (more details in the “Supplementary Material and Results” section). Under these circumstances, we did not test RiPP BGC-metabolite links, and we could not measure the performance of MetaMiner and DeepRiPP for this validation set; however, we tested both for the full metabolomes and genomes of these nine validation strains. In this test, MetaMiner annotated zero matches, despite the presence of 49 uncharacterized RiPP BGCs in the nine genomes. DeepRiPP “failed to load results,” even though the input data was correctly processed by other tools. On top of testing tools that connect mass spectrometry fragmentation data to BGCs, we also tested Nerpa (17) and GARLIC (18) which can connect structures (SMILES strings) to BGCs.

**Fig. 4.**
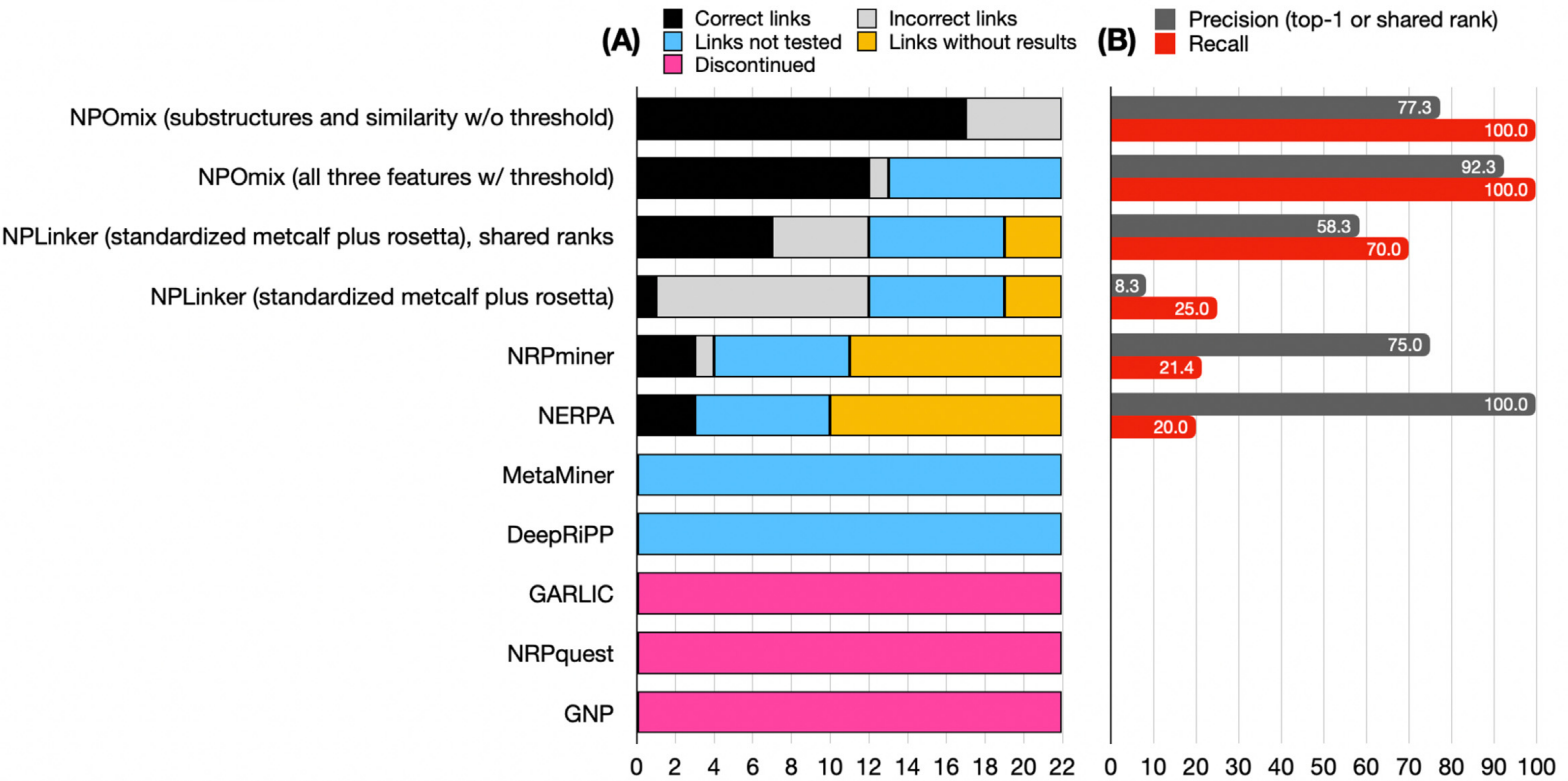
(A) Histogram displaying the number of correct links (true positives, in black), incorrect links (false positives, in gray), links not tested (due to limitations of the tool or threshold selected, in blue), and links without results (in yellow) for different currently available multi-omics tools. NPOmix, NPLinker, and NRPminer can link MS/MS spectra (known or cryptic) to BGCs; GARLIC and Nerpa can link known structures to BGCs. NPOmix has different versions that yield different precision scores and so does NPLinker, however, in this case, the scores were the same for the different versions. NPLinker has regular ranks and shared ranks (for example, from the 2nd to the 10th position, if all scores were equal, then all of them would be tied for second). The precision is the ratio between true positive links over all links that tested positives (true positives plus false positives), and this metric was calculated using only the best candidate (top-1) from the tools’ outputs. The recall is the ratio between true positives and true positives plus false negatives (links that were supposed to be true or negative positive results but were not classified as positives, in this case, links without results). NPOmix (all three features with co-occurrence threshold) had nine links below the threshold and these links were not tested. NRPminer and Nerpa can only test peptidic metabolites. GARLIC and GNP were discontinued (in pink), and they could not test any of the links. NRPquest was replaced by NRPminer (hence, also discontinued, in pink). There were no known RiPP metabolites in the validation set for MetaMiner and DeepRiPP to test; however, these tools did not generate results for the 49 unknown RiPP BGCs found in the genomes from the validation set. W/= with; W/o = without.

Fig. 4 is a histogram illustrating the performance of these tools (nature of each BGC-MS/MS link, precision and recall scores), including the two best versions out of eight versions of NPOmix displayed in Fig. S7 (four with and four without co-occurrence threshold). Recall is the ratio between the true positives over the true positives plus the false negatives (links that were supposed to be true or negative positive results but were not classified as positives, in this case, links without results). Fig. S8 exemplifies how fragmented BGCs can lead to scores below the threshold. More details on the Figs. S7 and S8 in the “Supplementary Material and Results” section. We note that we did not observe differences in NPLinker results using the standardized Metcalf score, the Rosetta score or both together. We selected top-1 accuracy to represent the precision scores since some tools only provide a single BGC candidate per metabolite tested. In theory, NRPminer and Nerpa should be able to test four links that are nonribosomal peptides (three orfamides and nevaltophin D) plus 11 additional hybrids between nonribosomal peptides and polyketides (two barbamides, five albicidins, two jamaicamides, cryptomaldamide, and antimycin). However, NRPminer could only generate predictions for four of these peptidic metabolites in the validated set (one incorrect and three correct links) and Nerpa only predicted three metabolites that were correctly linked to their BGCs. GARLIC can test all but four links (three pyocyanines and brasilicardin A) since it should work for nonribosomal peptides, polyketides, and hybrids. We were unable to compile GRAPE (tool required to use GARLIC) and we found an unanswered issue on their GitHub page with that same question, as well as another issue from 2016 that is left unanswered, thus it seems that this tool is also no longer supported or is discontinued.

NPLinker is systematic (it does not depend on the BGC/metabolite class) and, just like NPOmix without threshold, it can test all links. For all tools, we used standard settings (commands found in the notebook “NPOmix_SI-installation_and_running,” GitHub repository https://github.com/tiagolbiotech/NPOmix); and we note that for these analyses we did not attempt to optimize settings and scores of the various tools but used their (recommended) default settings. We also note that due to the use of a relatively small number of strains for the NPLinker benchmarking purposes (nine strains), it is likely that the correct links are part of a longer list of possible matches based on their co-occurrence score and that several links have the same score. Hence, the correct match may not be in the top-1 rank if we do not consider a shared rank (for example, from the 2nd to the 100th position, if all scores were equal, then all of them would be tied for second using their shared rank). The use of (many) more strains is likely to improve some rankings as the co-occurrence score is more likely to differentiate between different links. Comparing NPOmix to the *strain-correlation-based* (co-occurrence score) approach used within NPLinker we can see that: (i) the first tool uses a *k*-nearest neighbor approach that requires a training step (that can improve with more training paired data) while the second one does not need any training; (ii) the training step for NPOmix can process thousands of samples (paired genomes and metabolomes) in a scalable manner, while a *strain-correlation-based approach* requires much more processing power to run thousands of samples. The latter is mainly due to the fact that NPOmix selects which metabolites (MS/MS spectra) should be targeted for classification in opposition to NPLinker that attempts to link all metabolites present in the analyzed metabolomes; (iii) NPOmix is a tool for connecting metabolites to BGCs, NPLinker is a framework that facilitates integrative omics analysis, and in the future, NPOmix will likely become part of the NPLinker framework. We stress that “benchmarking NPLinker” effectively means benchmarking the (standardized) Metcalf strain-correlation and Rosetta scoring systems; in the future, it is likely a combination of various *strain-correlation-based* and *feature-based* scoring systems (such as NPClassScore) (39) that will enable the most effective integrative omics mining. Both precisions for top-1, and top-1 shared ranks are provided in Fig. 4. The complete results list with regular ranking and shared ranking for NPLinker can be found at Dataset S1, sheet six.

As illustrated in Fig. 4, the maximum number of correct links is 18 (17 from the MIBiG-GNPS validation set plus palmyramide, a new link partially validated) and this corresponded to using NPOmix with substructure and similarity scores without threshold and it presented a recall of 100% (no links were classified as false negatives). However, the highest precision (92.3%) could be found using NPOmix with all three features (similarity, substructures, and biosynthetic classes) with threshold and it also presented a recall of 100%. This top precision from NPOmix in our benchmarking experimentation was 34% more precise than the second-best tool tested, NPLinker with shared rank (Nerpa and NRPminer did present very high precision scores but their recall was fairly low because most of the tested links had no results), and NPOmix was able to correctly predict about 2.5 times more BGCs-metabolites links than NPLinker. Of note, the precision of NPLinker without shared rank was about 40% lower. Another difference between the most precise NPOmix version and the most precise NPLinker version was the recall, which NPOmix was 30% higher (100% versus 70%). As aforementioned, five tools could not be tested because they were: discontinued like GNP and NRPquest; generated no results like MetaMiner; had unclear instructions like GARLIC; or could not read the same input that other tools were able to process normally like DeepRiPP. We concluded from this figure that NPOmix not only can predict more links (Fig. 4A) than most of the other tools but also the ratio of correct links is significantly higher than other methodologies (Fig. 4B) and the recall displayed is optimal (100%, indicating the absence of false negatives). Of note, the prediction power of machine learning tools like NPOmix can be even higher with more top candidates (for this comparison we only used top-1) and better features, such as demonstrated by the implementation of biosynthetic class with NPClassScore (39) to improve NPLinker links and the use of extra features other than similarity by NPOmix. Additionally, the precision of NPOmix can improve with a bigger training set. In the future, we will compare NPOmix using the biosynthetic class feature with NPClassScore, since it would require standardizing and improving the current class predictions (using CANOPUS and MolNetEnhancer) for unknown/cryptic MS/MS spectra. Of course, an ideal NPOmix version would not only provide a perfect recall (100%) with a high precision (e.g. 92.3%), but also work for all links in the validation set, despite the use of a confidence threshold. We believe the current NPOmix represents a springboard for further development and improvements, also through integration with other mining tools.

### Using NPOmix and MassQL to computationally genome mine for new siderophores

The mining for specific specialized metabolites in a large number of metabolomics profiles is a challenging task. The available 3,248 LC-MS/MS chromatographic profiles obtained from the PoDP database would generate over 2.6 million MS/MS fragmentation spectra (representing measured ions via MS/MS fragmentation) if each file contains an average 800 spectra (as observed by inspecting some of these files). Post-dereplication, there are far too many new metabolites that can be isolated for natural product discovery. One way to subset spectra is by focusing on a particular type of activity. For example, siderophores can bind iron and, in some cases, can serve as an antibiotic by depleting iron levels in the media. MassQL (20) can detect whether a metabolite is bound to iron because its isotopic pattern is different from the *apo* (unbound) form and the delta *m/z* between the apo and the bound is equivalent to the mass for iron. By using MassQL on these 3,248 LC-MS/MS profiles (MassQL query listed in the “Methods” section), we detected only 380 putative siderophores (MS/MS fragmentation spectra, listed at Dataset S1, sheet seven), indicating a low recall and/or that siderophores are very rare in these samples. The latter is likely true because only 5.1% of the BGCs obtained from the PoDP database detected by antiSMASH are annotated as a siderophore. The putative siderophores identified by MassQL were filtered to remove redundancies and the final MGF format MS/MS spectra were used as input for NPOmix (MGF files can be obtained from the MassQL job listed in the “Methods” section).

NPOmix connected two of these siderophores with 100% Jaccard similarity (between BGC and MS/MS fingerprints) to BGCs labeled as siderophores by antiSMASH. The NPOmix version applied did not use biosynthetic classes or substructures for training (only similarity scores as features); given this, connecting metabolites bound to iron to siderophore BGCs increases the chances of true positives, since there is a very low probability of NPOmix matching the correct siderophore class randomly. The metabolite of [M-2H++Fe3+]+ *m/z* 780.918909 (hereafter *m/z* 780) was linked to the only siderophore BGC annotated by antiSMASH in the genome of the Burkholderiales *Achromobacter xylosoxidans* NH44784-1996 (GCA_000,967,095), indicating that NPOmix likely made a correct inference. Only one gene in the BGC linked to the *m/z* 780 has annotation (IucA/IucC family siderophore biosynthesis protein from the aerobactin BGC); however, aerobactin is not the closest MIBiG homolog (using a cutoff of 0.7, there is no homolog to this cryptic siderophore BGC). The gene cluster family for this candidate BGC had only two BGCs and the PoDP database average is six BGCs per family, highlighting the novelty of these BGCs. GNPS and Dereplicator+ had no library hits for the metabolite fragmentation spectra. Unfortunately, this sample was a cystic fibrosis bacterial isolate from the clinical laboratory, and it is no longer available to confirm a NPOmix prediction via isolation and structure elucidation. This pipeline can be reproduced by the users with their samples for prioritizing new siderophores out of many unknown metabolites.

## Discussion

In this work, we demonstrated the use of machine learning (a new *k*-nearest neighbors algorithm named NPOmix) and genome mining for processing several thousand LC-MS/MS files and about a thousand genomes to connect MS/MS spectra to GCFs. Our approach can systematically connect MS/MS spectra from known metabolites (links validated experimentally), spectra from metabolites analogous to known (links with GNPS library matches, exemplified by orfamide C), and spectra from cryptic metabolites (links without GNPS library matches and therefore absent from the MS/MS database, as exemplified by brasilicardin A). The advantage of using paired data is that the genomic information represents the full metabolic potential of an organism, and hence, we can prioritize the discovery of the most diverse naturally occurring BGCs via genome mining. In other words, novelty predictions using BGC-metabolite links are likely more comprehensive than just using metabolomics and a more accurate picture of the actual chemical diversity in nature. Additionally, the use of genetic information can help in the structure elucidation and prediction of bioactivity (2), highlighting the advantage of using the BGC information in the drug discovery process. Moreover, predicting linked MS/MS spectra for a promising BGC can facilitate their heterologous expression as the expression can be difficult if the target metabolite is not known. We also show how cryptic MS/MS spectra (absent from MS/MS databases like GNPS) can be annotated using NPOmix, MZmine (29), and Dereplicator+ (30), allowing expansion of the current MS/MS databases. Overall, NPOmix presents a new methodology to efficiently address the challenging task of connecting metabolites to BGCs and the results illustrated that this new method could impact future drug discovery pipelines since this kind of connection allows metabolomics to make use of *in-silico* predictions made via genomics (e.g., novelty, structures, and bioactivity).

The current focus of the NPOmix use was on finding BGCs for metabolites represented by their MS/MS spectra; however, NPOmix could also be used to go vice-versa. Also, despite this manuscript being centered on how paired data can make better *in-silico* predictions than just metabolomics, it could also be focused on how paired data is better than just genomics and the benefits would be very similar. We believe this strategy aims to be a pipeline that better uses the wealth of available data; therefore, it could maximize the chances of finding new drug-like metabolites.

We observed very good precision scores of top-1 = 81.8% and top-10 = 92.9% (both with randomness equal to 0, using the similarity and biosynthetic class features and using the Jaccard co-occurrence threshold). Additionally, we also obtained a good precision score of 77.3% even without the co-occurrence threshold (for *k* = 3 and all three kinds of features including similarity, biosynthetic class, and substructure predictions). We observed an annotation rate of around 28%, as 14 out of 50 MS/MS validated spectra were retained after the co-occurrence threshold. The annotation rate was even higher without the use of the co-occurrence threshold, 44% (22 out of 50 dereplicated MS/MS validated spectra). Table S1 details the number of genomes, metabolomes, BGCs, GCFs, and metabolites used in this study. More details on these 22 BGC-MS/MS links can be found in the Supporting Material (see the “Discussion” section) and Fig. S9.

In fact, compared to the other eight multi-omics tools, NPOmix seems to be the only available tool that is (i) systematic (suitable to many classes of BGCs), (ii) highly efficient (about 34% higher score in Fig. 4B than other approaches with 100% recall), (iii) high throughput (we processed around a thousand genomes and 3× more LC-MS/MS files), and (iv) scalable (requiring low-processing power, we used 8 MB of RAM and 8 cores). We note that NPLinker is also systematic, high throughput, and scalable; however, its precision score during benchmarking was 34% lower than NPOmix, in part due to the relatively low number of strains used during NPLinker benchmarking. NPOmix was able to correctly predict about 2.5 times more BGCs-metabolites links than other tools. The combination of multiple multi-omics tools (and other genomics and metabolomics tools) can be a potent genome mining approach that would enable the visualization of many kinds of predictions (similarity scores for the links, biosynthetic classes, complete/partial structures, taxa, and bioactivity) in a single “omics network” (work in progress), prioritizing new true positives. Here, it is also interesting to find out how the use of alternative mass spectral similarity scores, such as the recently developed machine learning-based Spec2Vec and MS2DeepScore (43, 44), will affect the performance of NPOmix. We also illustrated how combining NPOmix with MassQL can prioritize the discovery of putatively new siderophores, exemplifying the application of NPOmix for cryptic metabolites. This siderophore mining method can be easily reproduced by the NPOmix users if they search for metabolites bound to iron using the query string provided in the methods, extracting the MGF files (MS/MS spectra), running the NPOmix python version using only similarity as a feature and checking if NPOmix links a putative siderophore metabolite to a new siderophore BGC.

Of note, new links between metabolites with predicted structure and known BGCs may not mean that the metabolite fully corresponds to the predicted structure because mass spectrometry tends to not distinguish isomers and small changes in BGCs can yield substantial changes in the final structure. Therefore, NPOmix (a *correlation-based approach*) does not have the resolution to distinguish between isomers and regioselectivity of the various homologs. We would like to emphasize that new links (not experimentally validated links that are, for example, reported in the PoDP database) require proper elucidation to confirm the metabolite’s planar (2D) and absolute (3D) chemical structure. Hence, in this study, 6 of the 11 BGC-metabolite links (family of analogs) used for validation were previously fully elucidated (via knockouts, heterologous expression, and/or isolation and NMR structure elucidation) as recorded in the PoDP database. We want to emphasize that despite the small size of the validation set (11 BGC-metabolite links plus analogs, a total of 22 MS/MS spectra), we were able to combine *in-silico* tools like Mzmine, Dereplicator+, and NPOmix to create new links that can expand MS/MS mass spectral reference database as "level bronze" metabolites (new putatively annotated MS/MS spectra) (14). Interestingly, some of our *in-silico* structure predictions (such as for orfamides) were very accurate and they would substantially facilitate NMR structure elucidation of the naturally occurring metabolite.

Another aspect to mention is that the precision score may not be reproducible for every dataset, especially if the dataset is not too similar to the current training set, as they may present very different features; therefore, NPOmix features might not perform as well on a given atypical dataset. For example, by submitting a fungal species that has no BGC similar to any BGC in the NPOmix training dataset (obtained from the PoDP database), the output would contain no answer, since NPOmix requires similarity scores for classifying BGCs. Although, the insertion of even one additional fungal species to the training set that is similar to the submitted strain would cause the strain’s BGCs to network with training BGCs and NPOmix would generate outputs. Hence, by amplifying the training set (e.g., with the natural expansion of the PoDP database), NPOmix will be able to perform better for many different kinds of samples, including novel ones that have some similarity to strains in the training data. Unfortunately, none of the currents validated metabolites in the PoDP database belongs to the RiPP class (more details in the “Supplementary Material and Results” section); hence, we encourage specialized metabolism researchers to deposit examples of these types of ribosomal peptides (genomic and metabolomic data) to the existing PoDP database and these examples will be used in future tests.

The use of complete genomes over MAGs and metagenomes is preferred to create a more “complete” training set; we predict that this would result in better precision than if the training set is populated with several fragmented BGCs. It is important to clarify that some organisms do not have BGCs (e.g., higher plants and animals) and therefore their metabolites cannot be linked to biosynthetic genes using NPOmix. Regarding lower plants, the annotation of BGCs is now a hot topic; however, there is a current lack of paired data for plants and the current NPOmix demonstration is done on the suitable and available data from the PoDP (almost exclusively from bacteria). On top of that, BGC detection is more challenging for plants (e.g., more scattered biosynthesis genes).

Another challenging type of samples are microbiomes, such as nonaxenic cyanobacterial cultures like the example of the mix between *Moorea producens* JHB and its 15 detected heterotrophic genera plus viruses (45). Although, we successfully processed 40 cyanobacterial microbiomes and we linked five metabolites to their correct BGCs, demonstrating that this tool can also be used with microbiomes. A major limitation of using microbiomes for genome mining is that the quality of their metagenomes can hinder BGC assembly, leading to a smaller number of BGCs than expected and many of these BGCs can be fragmented. We plan to address microbiomes, plants, algae, and fungi samples more comprehensively in our future work (other future goals are also highlighted in the “Supplementary Material and Discussion” section).

Finally, we would like to stress that all true positive BGC-MS/MS validated links reported here were found in a known producer of the metabolites, they matched the reported masses, and 6 of the 11 BGC-metabolite links used for validation (true and false positives, including brasilicardin A) were reported at the PoDP database as validated via knockouts, heterologous expression, or isolation and/or NMR structure elucidation. In a similar way, NPLinker also used such validated links from the PoDP database to assess the impact of novel BGC-MS/MS linking scores and the complementary of them. Palmyramide A was also previously validated via isolation and NMR structure elucidation (46) (hence, making 7 out of 12 validated GCF-MS/MS links), and it will be soon published at MIBiG and PoDP. The putative orfamides were not confirmed via structure elucidation or co-elution with standards (the new metabolite spectra were annotated via GNPS library matching) but the comparison to a reference from GNPS and BGC amino acid predictions strongly indicates that they share the same planar structure as the reference metabolite from GNPS. Our results highlight the importance of making genomics and metabolomics data publicly available with curated metadata, because more available paired data would enable better training of models, and therefore, better tools for the research community.

## Conclusions

We developed a promising tool to search for new specialized metabolites in paired omics data of natural extracts by using links between cryptic MS/MS and cryptic biosynthetic gene clusters (BGCs) but also more efficiently dereplicating known BGCs or known metabolites. This will facilitate the use of genome mining in drug discovery pipelines. For example, we illustrated that our tool could integrate multiple kinds of information about biosynthetic classes, family beta-diversity, dereplication, and the probability of being a correct link. These bits of information are very useful for finding novel metabolites from nature (cultures or metagenomic samples). Thus, we believe that the current version of NPOmix will already facilitate access to the so far under-explored bacterial biosynthetic potential, allowing to bioprospect a larger portion of this potential.

Of note, we developed NPOmix and we were able to use other third-party tools like NPLinker (15), CANOPUS (35), NRPminer (13), Nerpa (17), GNPS molecular networking (14), BiG-SCAPE (7), and MetaMiner (10); these tools processed data from three databases (MIBiG, PoDP, and GNPS) (14, 20, 21). Interestingly, the information from linked BGC-MS/MS data enabled good predictions for partial structures like cryptomaldamide and even complete planar structures like orfamide C (including hints of the AA stereochemistry that can aid in the elucidation of absolute structures). Many of the links were known antifungal and antibiotic metabolites, therefore, reproducing this analysis for new metabolites can be a very promising methodology for drug discovery (work also in progress, especially focusing on the discovery of new siderophores, here exemplified by a computational approach including NPOmix and MassQL that can be reproduced by the users).

To facilitate the use of NPOmix, we are hosting workshops (in English and Portuguese) and we created video tutorials, both available at https://www.tfleao.com/npomix1. We will assist the NPOmix use so the genome mining community can benefit from its capabilities, especially via GitHub (https://github.com/tiagolbiotech/NPOmix_python for the python version or https://github.com/tiagolbiotech/NPOmix for the Jupyter notebook version).

We anticipate the use of this pipeline for many applications including (but not limited to): (1) studying siderophores and iron cycling under nutrient limitation; (2) studying humans, mammals, plants, marine invertebrates, and other microbiomes and their relationship with host health; (3) finding new bioactive metabolites for drug development; (4) better understanding metabolite-mediated cell function of a model organism like *E. coli* (important for heterologous expression); (5) learning how plants, corals, and phytoplankton can be more resilient to global warming and other anthropogenic impacts. In conclusion, we expect that our tool will play an important role to form an integrative omics mining community, and that it will have a range of implications for genomics, metabolomics, natural products discovery, and other associated research fields.

## Methods

See the “Supplementary Material and Methods” section for details on obtaining the data, assembly, BGC comparison, the NPOmix tool itself, and other bioinformatic analyses.

## Authors' Contributions

T.F.L. conceptualized the software; T.F.L., R.d.S., and As.B. programmed the software; M.W. and A.G. assembled the metagenomic reads and annotated all biosynthetic gene clusters; An.B. and R.R. worked on the predicted fragmentation for brasilicardin A; P.W.P.G. matched the predicted structure to the fragmentation of promising candidates and created structural insights; T.F.L., J.J.J.v.d.H., and M.W. curated the dataset and verified the known BGCs-metabolites links; T.F.L., J.J.R.L., J.J.J.v.d.H., and A.G. compared the performance of our tool with other multi-omics tools; E.G. cultured cyanobacterial samples and collected the cyanobacterial LC-MS/MS data published at PoDP database; J.J.J.v.d.H., H.W.K., R.R., A.T.A, P.W.P.G., and A.B. provided feedback on the approach and how to present the results; T.F.L. and J.J.J.v.d.H. wrote the manuscript; J.J.J.v.d.H., L.G., W.H.G., N.B., M.F.F., and P.C.D. funded and designed the research; L.G., W.H.G., N.B., M.F.F., J.J.J.v.d.H., and P.C.D. edited the manuscript; and all authors read, reviewed, and agreed to the published version of the manuscript.

## Preprint

https://www.biorxiv.org/content/10.1101/2021.10.05.463235.

## Supplementary Material

pgac257_Supplemental_Files

## Data Availability

The code (a collection of Jupyter notebooks) required to reproduce this work and to use the NPOmix tool for new samples can be found on the following GitHub repository page: https://github.com/tiagolbiotech/NPOmix. The repository also includes short video explanations on how the tool works and its importance for natural product discovery. We also wrapped the code in a few python scripts found here: https://github.com/tiagolbiotech/NPOmix_python. This python repository is the most updated version of the code, and it also contains more comments on each step of the pipeline. This page with the python version also includes a video explanation of how to prepare inputs and how to run NPOmix in python. Additionally, we provide a webpage (https://www.tfleao.com/npomix1) for submitting a limited number of samples for free processing (maximum of 50 queries MS/MS fragmentation spectra) and this page also includes workshops on how to submit samples and interpret results. We are going to purchase a server and allow users to submit many more metabolites than just 50. The (meta)genomes used to create the NPOmix training dataset for validation were downloaded from the PoDP (20) using notebook 1 from the GitHub repository (Jupyter notebook version). All BGCs from these training (meta)genomes can be found here for downloading: https://doi.org/10.5281/zenodo.6637083. The paired experimental MS/MS files were downloaded using the ftp links (also from the PoDP) found in Dataset S1, sheet eight. The links for these MS/MS files can also be found in the “NPOmix_SI-installation_and_running” notebook for downloading (GitHub repository https://github.com/tiagolbiotech/NPOmix). The testing set included MS/MS spectra from the PoDP database, spectra from the GNPS (14), and also spectra also used in the NPLinker publication (39). If the potential users find the tool challenging to run, we have our contact information on the main webpage (https://www.tfleao.com/npomix1) to submit samples and we expect that promising results will lead to fruitful collaborations.

## Notes

**Competing interest:** W.H.G. has an equity interest in NMRFinder and SirenasMD Inc., companies that may potentially benefit from the research results and W.H.G. also serves on the companies’ Scientific Advisory Boards. The terms of this arrangement have been reviewed and approved by the University of California San Diego in accordance with its conflict-of-interest policies. P.C.D. is a scientific advisor to SirenasMD Inc., Galileo and Cybele, and co-founder and scientific advisor to Ometa and Enveda with approval by the University of California San Diego. M.W. is a cofounder of Ometa Labs, LLC. J.J.J.v.d.H is a member of the Scientific Advisory Board of NAICONS Srl., Milano, Italy.
