# Supplementary material for: NPOmix: A machine learning classifier to connect mass spectrometry fragmentation data to biosynthetic gene clusters
Source: PNAS Nexus. 2022 Nov 16;1(5):pgac257. doi: 10.1093/pnasnexus/pgac257 (PMC9802219; doi:10.1093/pnasnexus/pgac257)
Supplement: pgac257_Supplemental_Files [file pgac257_supplemental_files.zip › PNASNEXUS-PNASNEXUS-2022-00689-T-s01.pdf]

NPOmix: a machine learning classifier to connect mass spectrometry fragmentation data to biosynthetic gene clusters

Authors: Tiago F. Leão[1,2], Mingxun Wang[1,3], Ricardo da Silva[4], Alexey Gurevich[5], Anelize Bauermeister[1], Paulo Wender P. Gomes[1], Asker Brejnrod[1], Evgenia Glukhov[6], Allegra T. Aron[1,7], Joris J. R. Louwen[8], Hyun Woo Kim[9], Raphael Reher[10], Marli F. Fiore[2], Justin J.J. van der Hooft[8,11**], Lena Gerwick[6], William H. Gerwick[1,6], Nuno Bandeira[1,3], Pieter C. Dorrestein[1,12,13,*].

Author Affiliations:
1 – Collaborative Mass Spectrometry Innovation Center, Skaggs School of Pharmacy and Pharmaceutical Sciences, University of California San Diego, La Jolla, California, USA.
2 – Center for Nuclear Energy in Agriculture, University of São Paulo, Piracicaba, Brazil.
3 – Center for Computational Mass Spectrometry, University of California San Diego, La Jolla, California, USA.
4 – NPPNS, Physic and Chemistry Department, School of Pharmaceutical Sciences of Ribeirão Preto, University of São Paulo, Ribeirão Preto, Brazil.
5 – Center for Algorithmic Biotechnology, St. Petersburg State University, St. Petersburg, Russia.
6 – Center for Marine Biotechnology and Biomedicine, Scripps Institution of Oceanography, University of California San Diego, La Jolla, California, USA.
7 – Department of Chemistry and Biochemistry, University of Denver, Denver, CO, 80210, USA.
8 – Bioinformatics Group, Wageningen University, Wageningen, the Netherlands.
9 – College of Pharmacy and Integrated Research Institute for Drug Development, Dongguk University, Gyeonggi-do, Korea.
10 – Institute of Pharmaceutical Biology and Biotechnology, University of Marburg, Marburg, Germany.
11 - Department of Biochemistry, University of Johannesburg, Johannesburg, South Africa.
12 – Center for Microbiome Innovation, University of California San Diego, La Jolla, California, USA.
13 – Departments of Pharmacology and Pediatrics, University of California San Diego, La Jolla, California, USA.

* Corresponding author. Email: pdorrestein@health.ucsd.edu.
** to whom correspondence should be addressed regarding the creation and management of the training data. Email: justin.vanderhooft@wur.nl.

Preprint: https://www.biorxiv.org/content/10.1101/2021.10.05.463235.
Classification: Biological Sciences – Microbiology.
Keywords: genomics; mass spectrometry; machine learning; specialized metabolites; biosynthetic gene clusters.

Supplementary Information

Background

Besides the fact that our validation set is small (11 BGC-metabolite links plus analogs, a total of 22 MS/MS spectra), we do show that the combination of *in silico* tools like Mzmine (1), Dereplicator+ (2) and NPOmix can create new links that can expand MS/MS databases as level bronze metabolites (new putative MS/MS spectra). Whilst >2000 validated BGCs are described in the MIBiG database, a minority of them have associated MS/MS spectra, and a subset of these can be expected to be present in samples under study for which actual paired omics data is available, explaining why the validation set is small). Nevertheless, we were able to correctly connect 17 known metabolites (including analogs) to their validated BGCs using their similarity, biosynthetic class, and substructure features. We also exemplified with orfamide C (peptidic metabolite) how the information from these links can yield very accurate *in silico* planar structure prediction with a low delta *m/z* (error between predicted and observed *m/z*). We believe the same approach can be used for new/cryptic metabolites since the KNN algorithm does not know the metabolite labels (e.g., orfamide C) while making the BGCs-metabolites links. Moreover, we could partially predict the stereochemistry of orfamide C and other metabolites using genomic data.

Generally, finding novel metabolites for cryptic BGCs or even known BGCs (e.g., new analogs) is very useful to accelerate natural product discovery, however, connecting known metabolites to their biosynthetic gene clusters is also important. Newly linked BGCs for known metabolites can lead to the discovery of new enzymatic processes. For example, in the strain *Anabaena variabilis* ATCC 29413, a nonribosomal peptide-synthetase (NRPS) gene is responsible for the attachment of a serine residue to generate the final mycosporine-like amino acids (MAA) product. However, in the strain *Nostoc punctiforme* ATCC 29133, this same step is performed by an ATP-grasp ligase (3). This highlights that different microbes can generate the same metabolite through different convergent biosynthetic routes.

Results

Comparing the performance of NPOmix with other published tools. Capistruin C was “bioinformatically inferred through comparative analysis with another experimentally defined gene cluster”. Therefore, capistruin C was not validated in the reported strain at PoDP (*Burkholderia thailandensis* E264). The strain in which the BGC was validated is absent from the MassIVE database (*Burkholderia thailandensis* E444), meaning that we could not obtain the metabolome required for testing this strain. Moreover, we believe that this prediction “inferred through comparative analysis” does not need to be correct because the *B. thailandensis* E264 genome was included in the NPOmix analysis but the MIBiG BGC did not network with any other BGC in the full dataset (including the MIBiG BGC known for producing capistruin C), and we also checked the *B. thailandensis* E264 antiSMASH result for a capistruin C annotation but, again, none of its BGCs had the capistruin C annotation as the “most similar known cluster”. We

were unable to find the MS/MS spectra for polytheonamide A and B, they are not published at GNPS.

As observed in Fig. S7A, the absolute number of correct BGC-metabolite links is bigger for NPOmix using the substructure and similarity features without the co-occurrence threshold compared to the same condition using the co-occurrence threshold, because some links are present in several samples and some BGCs can be silent or very fragmented. This typically creates mismatches between the fingerprints and drops the Jaccard index in the presence/absence of the fingerprints used for the co-occurrence threshold. However, some not so fragmented BGCs can be connected via BiG-SCAPE similarity to a reference BGC and then get grouped into the same family, see for example in Fig. S8. Additionally, in some samples, the MS/MS spectra might not be acquired due to the complexity of the sample and the nature of the data-dependent acquisition. Hence, in these cases, the co-occurrence threshold may remove links (illustrated as "links not tested" in Fig. S7A and Fig. 4) even if they are true positives (metabolite correctly linked to its BGC). For NPOmix using similarity only and using both similarity and biosynthetic class, the threshold only removed false positives. Despite the higher number of correct links without the threshold, the threshold increases the relative ratio of correct links, leading to higher precision scores if compared to the NPOmix versions without threshold (Fig. S7B).

Discussion

We created a machine learning solution, a k-nearest neighbors algorithm named Natural Products Mixed Omics (NPOmix) approach, to connect natural products observed by untargeted mass spectrometry to their corresponding biosynthetic gene clusters (BGCs). We showed that a large dataset, deriving from heterogeneous sources such as the ones currently available in the Paired Omics Data Platform (PoDP), can create good fingerprints and can thus successfully connect known metabolites to their corresponding BGCs, such as albicidin and its analogs to a BGC in *Xanthomonas albilineans* GPE PC73 (GenBank ID GCA_000087965.1), orfamides A-C to a BGC in *Pseudomonas protegens* Pf-5 (GCA_000012265), and cryptomaldamide and jamaicamide A and C to BGCs in *Moorena producens* JHB (GCA_001854205). All three of these strains were the original producers of these metabolites. In Fig. S4, we illustrated how the BGC predictions (such as predicted moieties) can help to prioritize true links over false positives via comparing predicted structures between a given MS/MS spectrum and its BGC candidates (the matching was done using only similarity and biosynthetic class as features for the NPOmix algorithm).

We are developing an integrated pipeline for metabolite discovery using genomics, LC-MS/MS metabolomics, and other multi-omics tools. Additional future work will include the testing of other similarity metrics for networking and fingerprinting such as BiG-SLICE (4) for genomics and Spec2Vec (5) and MS2DeepScore (6) for the metabolomics. We will also look for synergy with correlation scores from NPLinker (7) to better annotate paired omics datasets. We intend to implement biosynthetic class and substructure predictions straight from the MS/MS fragmentation spectra using tools like SIRIUS 4 (8), MS2LDA (9), MolNetEnhancer (10), or CANOPUS (11), prioritizing candidates that have several substructures or predicted classes matching between BGCs and MS/MS spectra. The GNPS molecular family information could be

used to select a consensus prediction among different MS/MS spectra from the same family. Enrichment of the current Paired Omics Data Platform dataset (we could now use 1,040 PoDP samples for NPOmix) with higher quality samples as well as more validated BGC-MS/MS links will further drive the development of tools such as NPOmix, and this will spark the discovery of more novel NPs.

We intend to add to this multi-omics approach: 1) BGC bioactivity (12); 2) MS/MS bioactivity, by creating a new machine learning tool to predict bioactivity straight from the MS/MS spectra, and; 3) MS/MS substructure predictions, by integrating tools like MS2LDA (9), CSI: FingerID /SIRIUS 4 (8) and MassQL (13). NPClassScore (14) demonstrated that biosynthetic class can be predicted with a combination of CANOPUS and MolNetEnhancer, hence, the NPOmix users can already run the KNN version with similarity and biosynthetic class as features, a version that yielded a precision of 92.9% and a recall of 100% in the validation set. Accurate *in silico* structure and bioactivity predictions (only possible by using an efficient method to connect metabolites to BGCs and access these *in silico* genomic predictions) allow to start using a more "modeling" way to investigate all sequenced environmental bacteria/microbiomes, reducing the need for multiple cell assays and significantly aiding structure elucidation.

NPOmix was able to correctly link nine out of 12 different known metabolites (18 out of 23 MS/MS spectra, including analogs) to their corresponding BGCs. These metabolites (and analogs) were barbamide, antimycin A1, pyocyanine, brasilicardin A, orfamide A-C, albicidins, jamaicamide A and C, cryptomaldamide and palmyramide A (the last one was tested separately from the validation set and it was not used to estimate precision scores in Fig. 3 and 4). These known metabolites were reported from 9 strains belonging to the phyla Proteobacteria (44.44%), Actinobacteria (33.33%), Cyanobacteria (22.22%), and from seven different genera (Fig. S9A), indicating taxonomic diversity in this bacterial validation dataset. We can also see in Fig. S9B that these links encompass distinct biosynthetic classes like polyketide synthase (PKS), nonribosomal peptide-synthetase (NRPS), hybrid PKS-NRPS, hybrid terpene-oligosaccharide, and other; indicating that the tool is indeed systematic. Lastly, most of these links were reported to be active against fungi and bacteria (Fig. S9C), suggesting that NPOmix can link metabolites to BGCs with promising bioactivity.

Methods

Obtaining paired data. The paired data used in this manuscript is available at the Paired omics Data Platform (PoDP) and we were able to obtain 36 out of 71 meta-datasets that were available at the time. We automatically downloaded the paired (meta)genomics-metabolomics data from the samples in the PoDP according to the code in notebook 1 at the GitHub repository described below.

Metagenome assembly and annotation, BGC, and MS/MS similarity calculation. Metagenomic reads were assembled with SPAdes 3.15.2. (15). For BGC annotation, we used antiSMASH 5.0 (16) and for gene cluster networking we used BiG-SCAPE 1.0 (similarity cutoff of 0.7)(17). BiG-SCAPE raw distance is measured via the domain sequence similarity (DSS) index, an index that calculates the Pfam domain copy number differences and sequence identity. For networking

metabolites, we used GNPS classical molecular networking release 27 (similarity cutoff of 0.7). We did not use the full classical molecular networking capabilities in the NPOmix approach, as only the functions required to calculate a modified cosine score between a pair of MS/MS spectra were needed.

Creating fingerprints. We developed python scripts and we combined them with scripts from sklearn (https://scikit-learn.org/stable/index.html) to create both BGC and MS/MS fingerprints and to run the KNN algorithm. A BGC fingerprint is created by pairwise BiG-SCAPE comparison between the queried BGC and all the BGCs found in the (meta)genomes in the training set, selecting the highest similarity scores for each (meta)genome. An MS/MS fingerprint (part of the testing set) is created by pairwise modified cosine comparison between the queried MS/MS and all the MS/MS present in the LC-MS/MS files paired with the genomes from the training set, also selecting only the highest similarity scores per set of experimental MS/MS spectra.

Jupyter notebooks. All scripts used in this research can be found at this GitHub repository: https://github.com/tiagolbiotech/NPOmix. For instructions on installing and running the tools used in this publication, please see "NPOmix_SI-installation_and_running". Notebook 1 can be used to download (meta)genomes and metagenome-assembled genomes (MAGs) that contain paired untargeted metabolomics (LC-MS/MS)(metabolomic files will also be downloaded by the notebook). We selected genomic samples that contained a valid Genome ID or BioSample ID, resulting in 732 genomes/MAGs. We also selected and assembled 1,034 metagenomes. Notebook 2 can be used to process downloaded metabolomics files and a selected set of ".mgf" reference MS/MS spectra, creating a matrix containing the MS/MS fingerprints for the selected set of reference spectra (reference MS/MS spectra for the validation but for using the tool these reference spectra will be replaced by cryptic MS/MS spectra). If there were more than one LC-MS/MS file per genome (for example different media conditions or different chemical fractions), these files were merged into a single file representing these experimental MS/MS spectra. Notebook 3 can be used to process the antiSMASH results to create BGC fingerprints and use those to train the KNN algorithm. The MS/MS fingerprints are used to predict a/multiple GCF(s) for each tested reference MS/MS spectra found in the paired genomes-MS/MS data. We filtered the GCF-MS/MS links for cases in which the top GCF candidate had co-occurrence with a cutoff of 0.7 (GCF and MS/MS scores were present in the same set of samples (the cutoff is the same as the similarity cutoffs for consistency). Notebook 3 also performs cross-validation (dividing the data into 5 parts) and the average precision score for the cross-validation was 56.9%. Notebook 4 can be used to generate metadata such as the type of GCF or the count of BGCs per each genus in the database. Notebook 5 presents the code for genome mining that yielded the annotation of brasilicardin A (more details below). Notebook 6 expanded the similarity/absence fingerprints by including the biosynthetic class as new features, notebook 7 included the substructure predictions as new features and notebook 8 uses all three features (similarity, biosynthetic class, and substructures).

Genome mining for new MS/MS spectra using Dereplicator+ and NPOmix. In order to use the NPOmix approach to find new NPs without any GNPS library matches (absent from the MS/MS database), we developed a pipeline combining NPOmix, MZmine (1), and Dereplicator+ (2).

First, several strains can be selected using MZmine, here exemplified with 16 strains, based on their BGC beta-diversity scores. The Jaccard beta-diversity score metric of the similarity between a pair of strains was calculated as the intersection over the union of the detected gene cluster families. Using MZmine, we select peaks that were above a certain intensity threshold (we used base peak relative abundance of 1E6) to prioritize the chromatographic peaks that could reasonably be isolated for structure elucidation. In this example, we detected approximately 3,800 peaks with MS/MS spectra found in the analysis of the 16 most diverse strains. This MZmine list of peaks that have associated MS/MS data was filtered for a minimum precursor mass of *m/z* 500 to promote the presence of multiple moieties (substructures) in the predicted structures, generating 300 ".mgf" files. These mgf files were used by NPOmix to predict the GCFs/BGCs for each of the 300 MS/MS spectra. We filtered for BGC-MS/MS links that the query MS/MS spectra existed in the same strains that the query BGCs were found and not across different strains, using the Jaccard index in the presence/absence of the fingerprints, essentially a pairwise analysis between the BGC fingerprint and the MS/MS fingerprint. This second filter narrowed down the number of mgf files to 72. These 72 mgf files were processed by Dereplicator+ for predicting structures for each MS/MS spectrum, leading to the annotation of brasilicardin A. Two other Dereplicator+ hits did not match the predicted GCFs. MZmine parameters were as follows: noise level of 1E6 for MS1 and 1E3 for MS/MS, minimum group size in the number of scans of 4, group intensity threshold of 1E6, the minimum highest intensity of 3E6, *m/z* tolerance of 10 ppm, retention time tolerance of 0.2, weight for *m/z* of 75%, and weight for a retention time of 25%.

Expanding BGC and MS/MS fingerprints using biosynthetic classes. In notebook 6, the BGC classes were annotated and included in the BGC fingerprints. To accomplish this, all of the antiSMASH annotations for a given BGC were added to the presence of all predicted classes. Each class represented a new column in the fingerprints and the columns were filled with 1 (if the class was present) and 0 (if the class was absent). We observed the following classes in our dataset: polyketide synthases, nonribosomal peptide-synthetases, terpenes, siderophores, ribosomally synthesized and post-translationally modified peptides, phosphonates, oligosaccharides, phenolic metabolites, others/unknowns, other minor classes (aminoglycoside/aminocyclitol, beta-lactone, beta-lactam, butyrolactone, ectoine, furan, fused, indole and phenazine), and combinations of more than one class (like PKS-NRPS). We added in total 10 new presence/absence biosynthetic features. In the MS/MS fingerprint, for each one of the 22 validated MS/MS spectra, we annotated the presence/absence of the biosynthetic classes based on the known structures. These new fingerprints were used in the machine learning process, analogously to the notebook 3.

Expanding BGC and MS/MS fingerprints using substructure prediction. The substructure predictions were also annotated using antiSMASH and they were implemented in the BGC fingerprints. Analogously to the biosynthetic classes, each substructure prediction represented a new column in the fingerprints and the columns were filled with 1 (if the class was present) and 0 (if the class was absent). We annotated 85 substructures (the substructures found in our samples are listed in Dataset S1, sheet five) and these substructures were added as 85 new features. We also tested summarizing and PKS substructures (malonyl-CoA, methylmalonyl-

CoA, and so on) and all KR domains into two columns (one for each). Regarding the MS/MS fingerprints for each of the 22 validated MS/MS spectra, we annotated the columns based on the information from the known BGCs. For palmyramide A we manually annotated the substructures from its known structure. The new BGC and MS/MS fingerprints were used in the machine learning algorithm to calculate precision scores. Predicted structures were obtained by analyzing the substrate-specific from the antiSMASH BGCs and manually matching them to the MS/MS fragmentation spectra.

Benchmarking multi-omics tools (comparing the performance of NPOmix with other tools). We attempted to execute eight multi-omics tools for benchmarking NPOmix: MetaMiner (18), DeepRiPP (19), NRPquest (20), NRPminer (21), GNP (22) and NPLinker (7), Nerpa (23) and GARLIC (24). For the sake of computational resources, we only performed the benchmarking on the 9 strains that contained the 22 BGC-metabolite-structure links used for NPOmix validation and for calculating the NPOmix precision scores. We selected standard configurations for all these eight multi-omics tools and, unfortunately, we were unable to run DeepRiPP (could not process the FASTA files), NRPquest (discontinued), GNP (discontinued), and GARLIC (we were unable to compile its pre-requirement, GRAPE, and it seems that this tool was also discontinued). MetaMiner produced no results, despite the presence of 49 unknown RiPP BGCs in these 9 samples.

Detecting siderophores with MassQL. We used the MassQL (13) version 31.4 and the job can be found here: https://proteomics2.ucsd.edu/ProteoSAFe/status.jsp?task=2380461c4a624c39b7b19a4f955515e7. In this job, we used the following query "QUERY scaninfo(MS2DATA) WHERE MS1MZ=X-1.993:INTENSITYMATCH=Y*0.063:INTENSITYMATCHPERCENT=25:TOLERANCEPPM=10 AND MS1MZ=X:INTENSITYMATCH=Y:INTENSITYMATCHREFERENCE:INTENSITYPERCENT=2 AND MS1MZ=X+1.0034:INTENSITYMATCH=(Y*X*0.04911/100+Y*1.2397/100):INTENSITYMATCHPERCENT=20:TOLERANCEPPM=10 AND MS1MZ=X-52.91:TOLERANCEPPM=10 AND MS2PREC=X-52.91". This query searches to the MS isotopic pattern that characterizes metabolites bound to iron and this metabolite needs to contain a MS/MS fragmentation spectrum. Then, it also looks for the unbound version (X-52.91) that also contains a MS/MS fragmentation spectrum. The outputs were filtered for condensing duplicates with defined tolerance using a simple python code and the final outcome consisted of 380 putative siderophores (listed at Dataset S1, sheet seven).

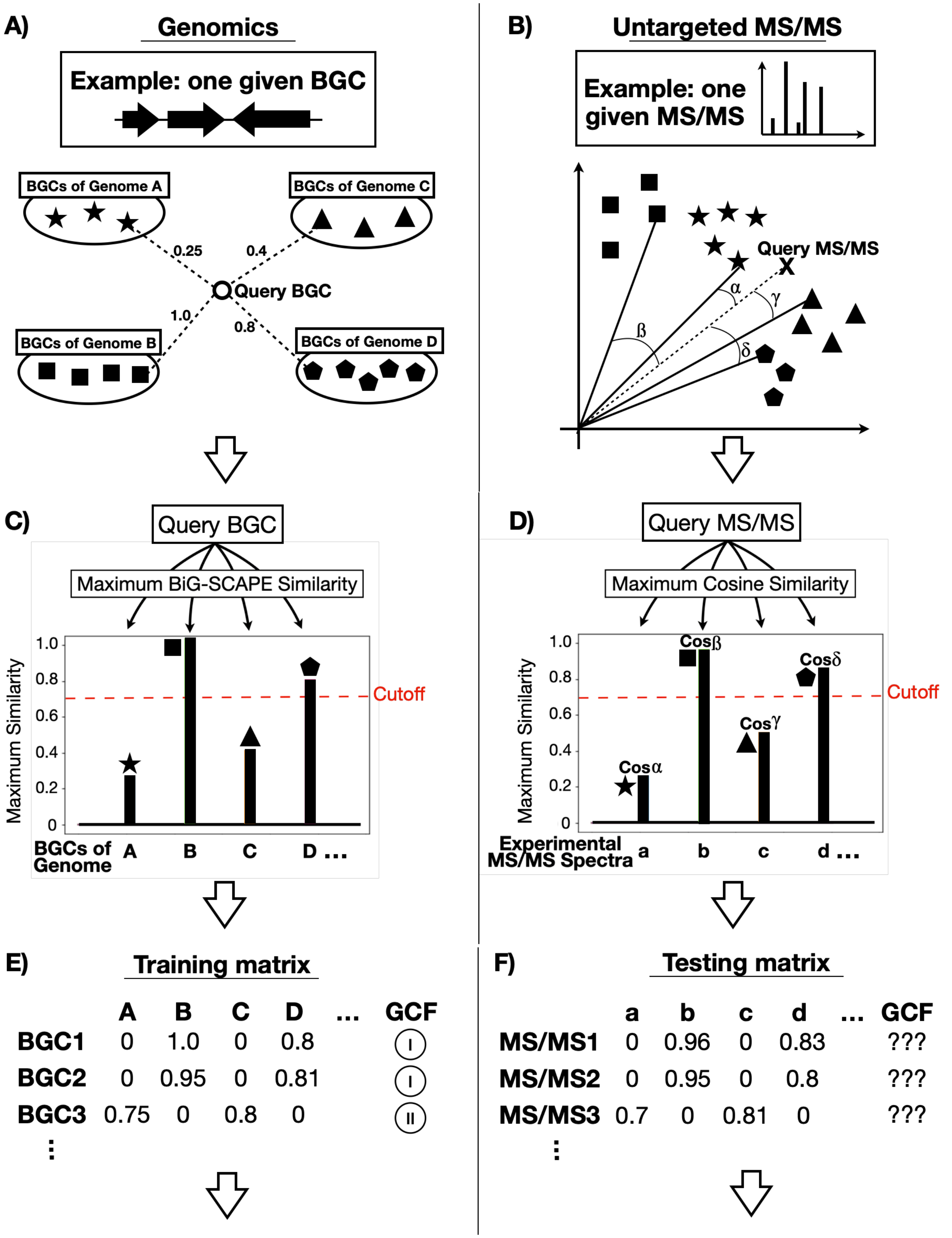
A)
Genomics
Example: one given BGC
BGCs of Genome A
BGCs of Genome C
0.25
0.4
Query BGC
1.0
0.8
BGCs of Genome B
BGCs of Genome D
B)
Untargeted MS/MS
Example: one given MS/MS
Query MS/MS
X
α
γ
β
δ
C)
Query BGC
Maximum BiG-SCAPE Similarity
Maximum Similarity
1.0
0.8
0.6
0.4
0.2
0
Cutoff
BGCs of Genome
A
B
C
D ...
D)
Query MS/MS
Maximum Cosine Similarity
Maximum Similarity
1.0
0.8
0.6
0.4
0.2
0
Cosβ
Cosδ
Cosγ
Cosα
Cutoff
Experimental MS/MS Spectra
a
b
c
d ...
E)
Training matrix
A B C D ... GCF
BGC1 0 1.0 0 0.8 I
BGC2 0 0.95 0 0.81 I
BGC3 0.75 0 0.8 0 II
⋮
F)
Testing matrix
a b c d ... GCF
MS/MS1 0 0.96 0 0.83 ???
MS/MS2 0 0.95 0 0.8 ???
MS/MS3 0.7 0 0.81 0 ???
⋮

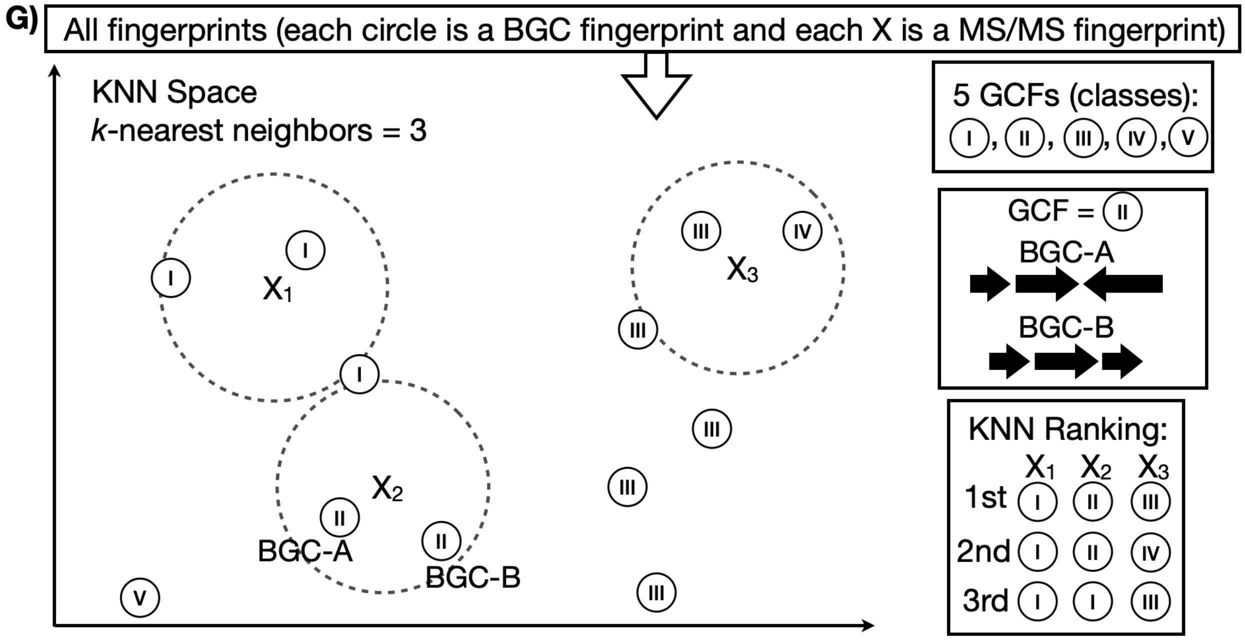

Fig. S1. The genomics and metabolomics pipelines use the proposed KNN approach for a hypothetical dataset with 4 paired genomes-MS/MS samples. Representation of how to calculate the similarity scores between BGCs (displayed in A) and between MS/MS spectra (displayed in B). Schematic of how to create BGCs (C) and MS/MS (D) fingerprints using a paired genomics-metabolomics dataset of four samples, e.g., genomes, metagenomes, or MAGs (samples A-D) and similarity scores from BiG-SCAPE and GNPS. The dashed red line represents the selected cutoff of 0.7. The BGC1 is highly similar to a BGC in sample B (score of 1.0 indicating an identical BGC), while it is probably absent in samples A and C. The BGC fingerprints are grouped together in a training matrix and the MS/MS fingerprints compose the testing matrix (F). All fingerprints are plotted in the multi-dimensional KNN space (G, here represented in only 2D for simplification) where each circle represents a BGC fingerprint, and each X represents an MS/MS fingerprint. BGCs are labeled according to one of the five GCFs (five differently colored circles). KNN ranking of neighbors is based on the proximity between the testing MS/MS fingerprint and the neighboring BGC fingerprints. In this example, a KNN = 3 (three closest neighbors) is depicted. BGC = biosynthetic gene cluster; MS/MS = mass fragmentation spectrum; KNN = *K*-nearest neighbor; BiG-SCAPE = software to calculate pairwise BGC-BGC similarity; GCF = gene cluster family (group of similar BGCs); Cosine score = modified cosine score from GNPS to calculate pairwise spectrum-spectrum similarity. For example, the circle with “I” inside that is shared between X_1 and X_2 could represent a lipopeptide that shares the peptidic portion with other “I” circles and a fatty acid portion with the “II” circles or vice versa.

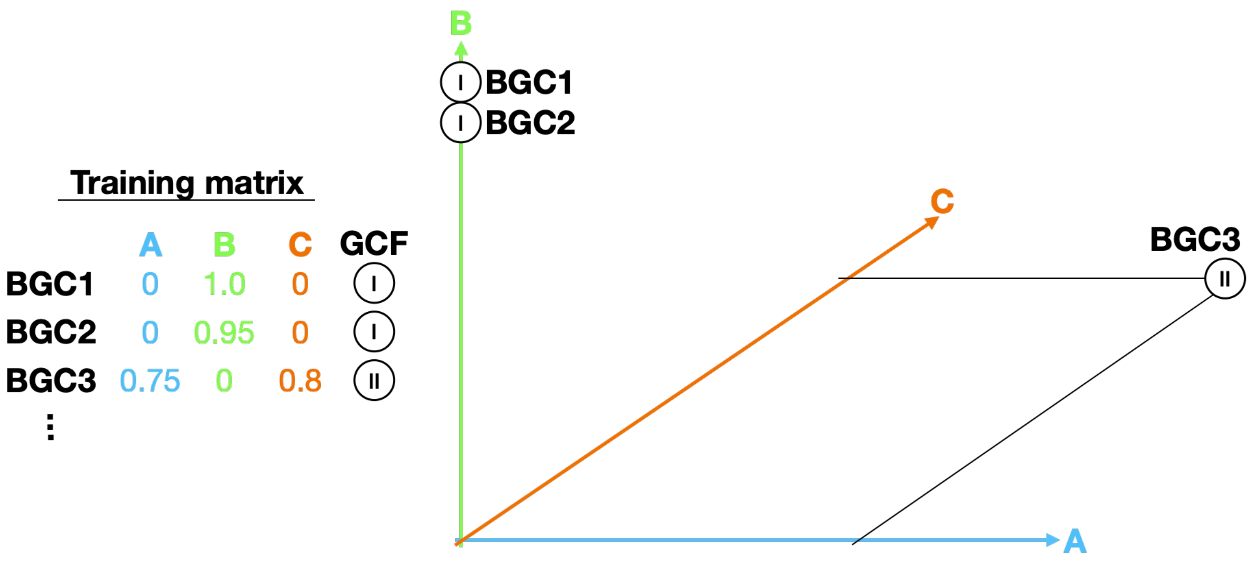

Fig. S2. Representation of how BGCs can be plotted in the KNN (*k*-nearest neighbor) space by using the values in the training matrix, each column represents a genome in the training set and it also represents a dimension in the KNN space (1,040 genomes distributed in 1,040 columns). This example has three dimensions because it uses only three genomes; the actual training matrix used in this study had 1,040 genomes and therefore 1,040 dimensions. BGC = biosynthetic gene cluster; GCFs = gene cluster family.

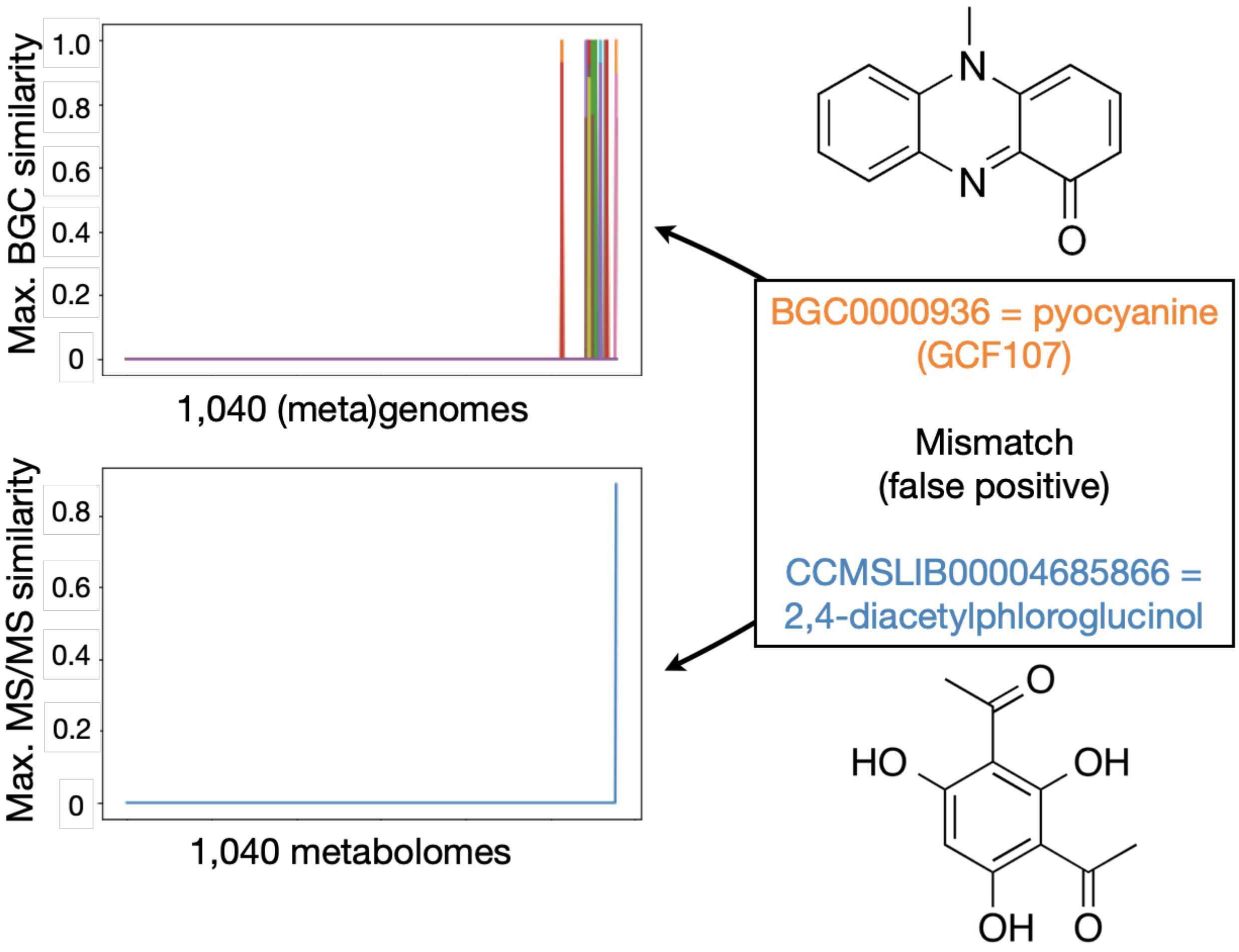

Fig. S3. Representation of a mismatch linked by the KNN algorithm using *k* = 3. The closest neighboring BGC fingerprint for pyocyanine does not properly match the MS/MS fingerprint from the metabolite 2,4-diacetylphloroglucinol because none of the peaks in both fingerprints overlap (in other words, none of the BGCs in the top fingerprint belongs to the same strain that the metabolite 2,4- diacetylphloroglucinol is present), indicating that NPOmix suggested the wrong GCF for the 2,4- diacetylphloroglucinol MS/MS spectrum. BGC = biosynthetic gene cluster; GCFs = gene cluster family.

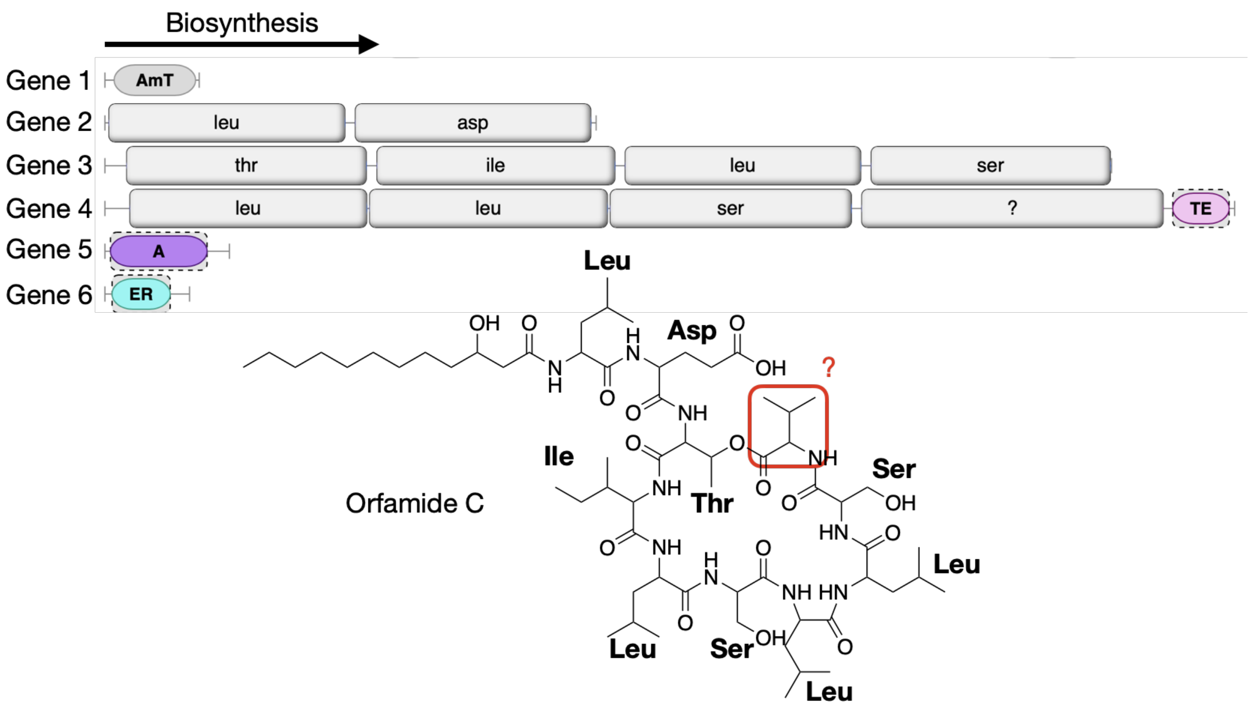

Fig. S4. NPOmix automatically connected an MS/MS spectrum annotated as "putative orfamide C" to the MIBiG BGC annotated as orfamide C. The figure illustrates the matches between the BGC's AA predictions (via antiSMASH) and the predicted metabolite structure (orfamide C, predicted via MS/MS spectral matching). Only one AA (valine, in red) out of 10 AA could not be predicted by the BGC annotation tool (antiSMASH), however, this valine residue was predicted by the MS/MS spectrum. BGC = biosynthetic gene cluster; AA = amino acid; AmT = aminotransferase; TE = thioesterase; A = adenylation domain; ER = enol reductase; "?" in the BGC represents that one AA could not be predicted by antiSMASH.

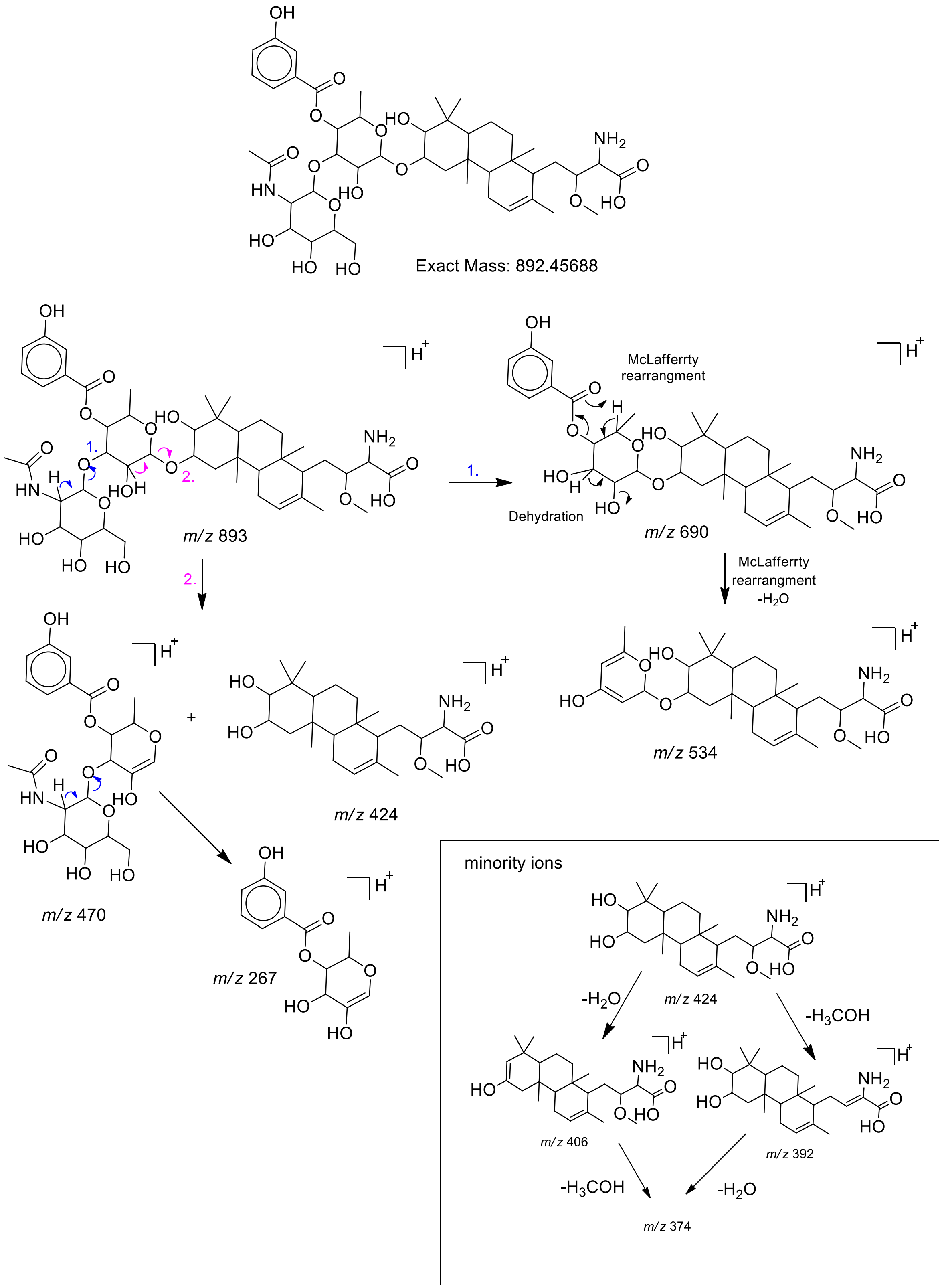
Exact Mass: 892.45688
m/z 893
1.
2.
McLafferrty rearrangment
Dehydration
m/z 690
McLafferrty rearrangment -H2O
m/z 534
m/z 424
m/z 470
m/z 267
minority ions
m/z 424
-H2O
-H3COH
m/z 406
m/z 392
-H3COH
-H2O
m/z 374

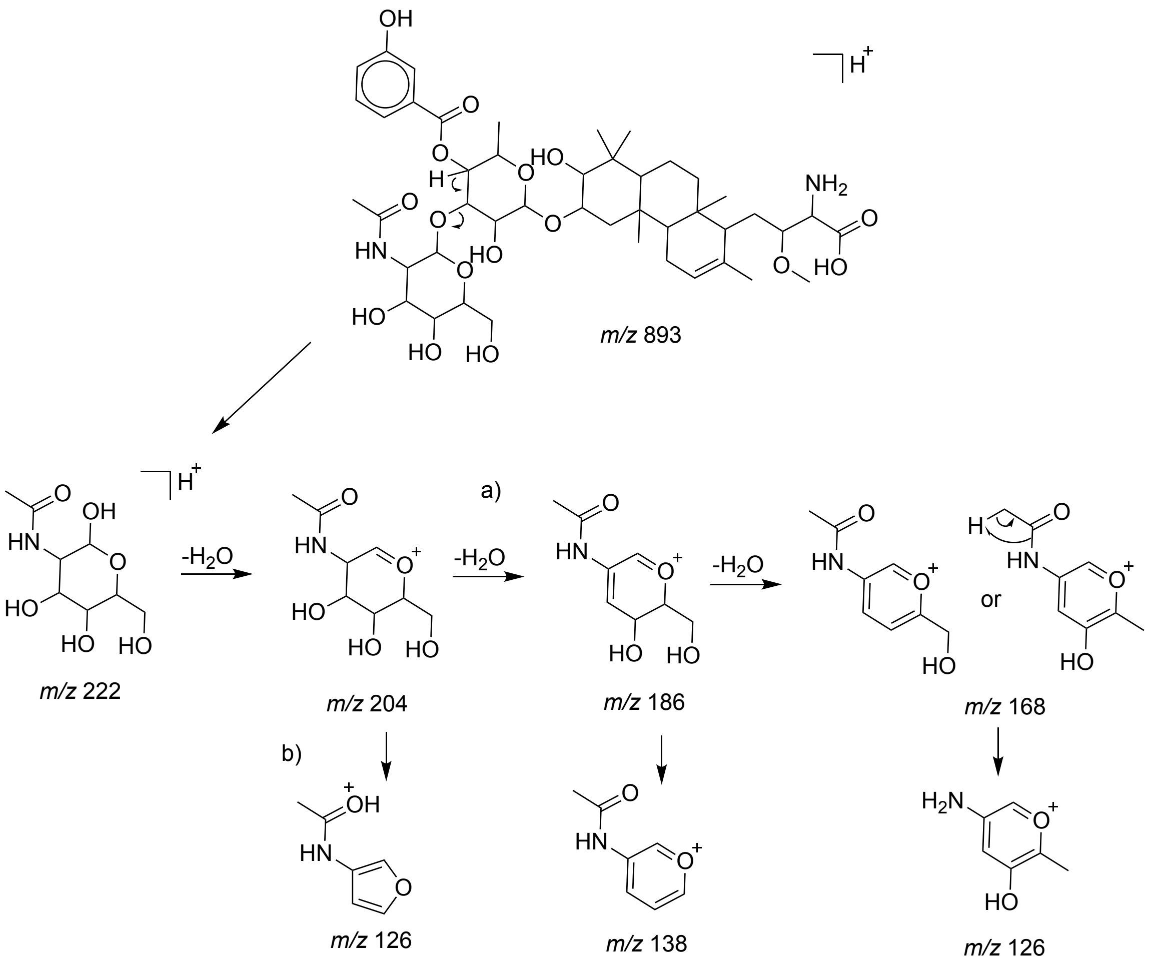

Fig. S5. Proposed mechanism for the fragmentation of brasilicardin A by ESI mass spectrometry. The structure was proposed by NPOmix as a possible match for the MS/MS spectrum with protonated *m/z* 893.4624. Dataset S1, sheet four, shows the SMILES strings and delta *m/z* values for the predicted structural fragments and the observed fragments in the MS/MS spectrum. All delta *m/z* values in the table were extremely small, strongly indicating that brasilicardin A is the correct structure for this MS/MS spectrum and it matches well with the BGC identified in the genome of *Nocardia terpenica* IFM 0406 (BGC known to produce brasilicardin A, ID BGC0000632).

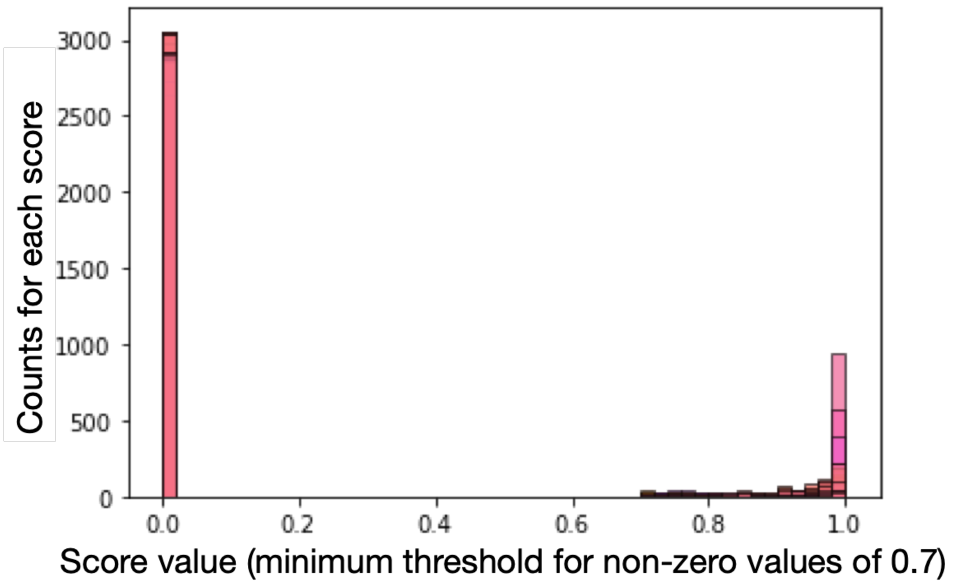

Fig. S6. The distribution of the training dataframe, including all three kinds of features (similarity scores, biosynthetic classes, and substructure predictions). Minimum threshold for non-zero values was 0.7 and scores for biosynthetic classes and substructure predictions were binary (either 0.0 or 1.0).

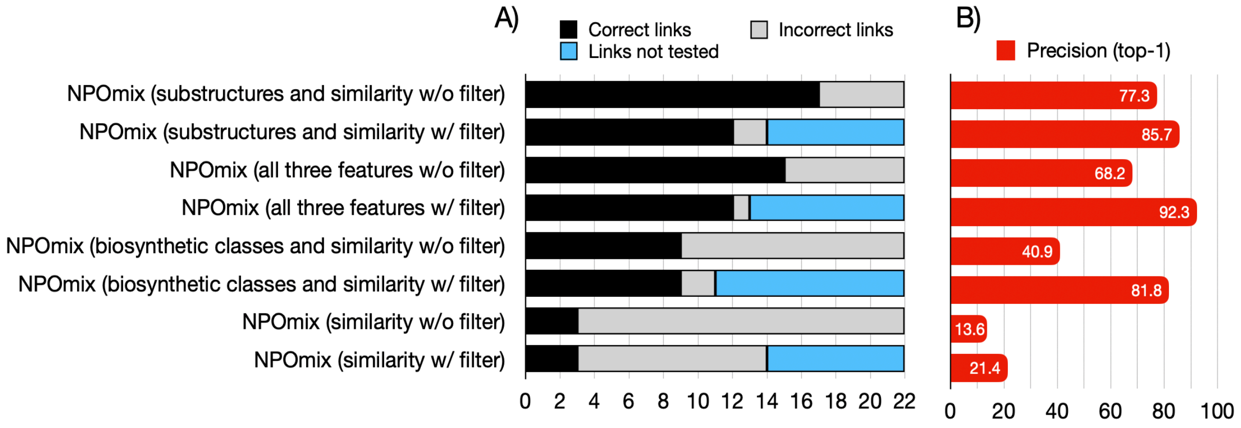

Fig. S7. A) Histogram displaying the number of correct links (true positives, black), incorrect links (false positives, grey), links not tested (due to limitations of the tool or threshold selected, light blue) for different currently available multi-omics tools.

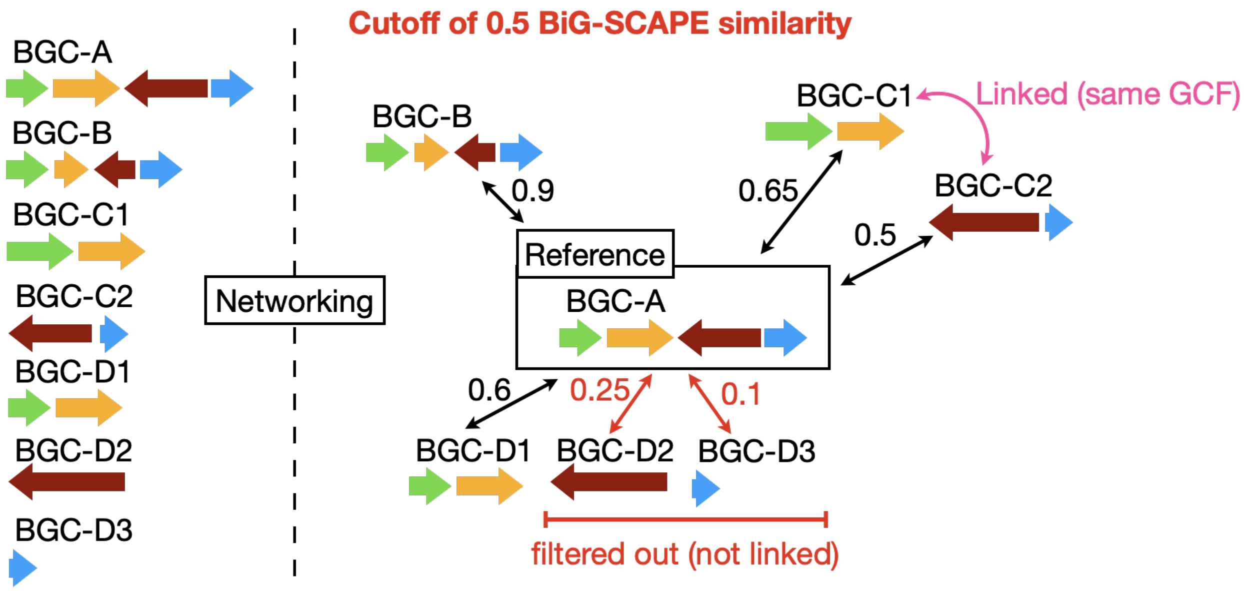

Fig. S8. Example of fragmented biosynthetic gene clusters (BGCs) where the fragments are connected by BiG-SCAPE similarity, grouping them in a gene cluster family (in some cases). In other cases, the partial BGC still matches the reference, or some fragments are networked.

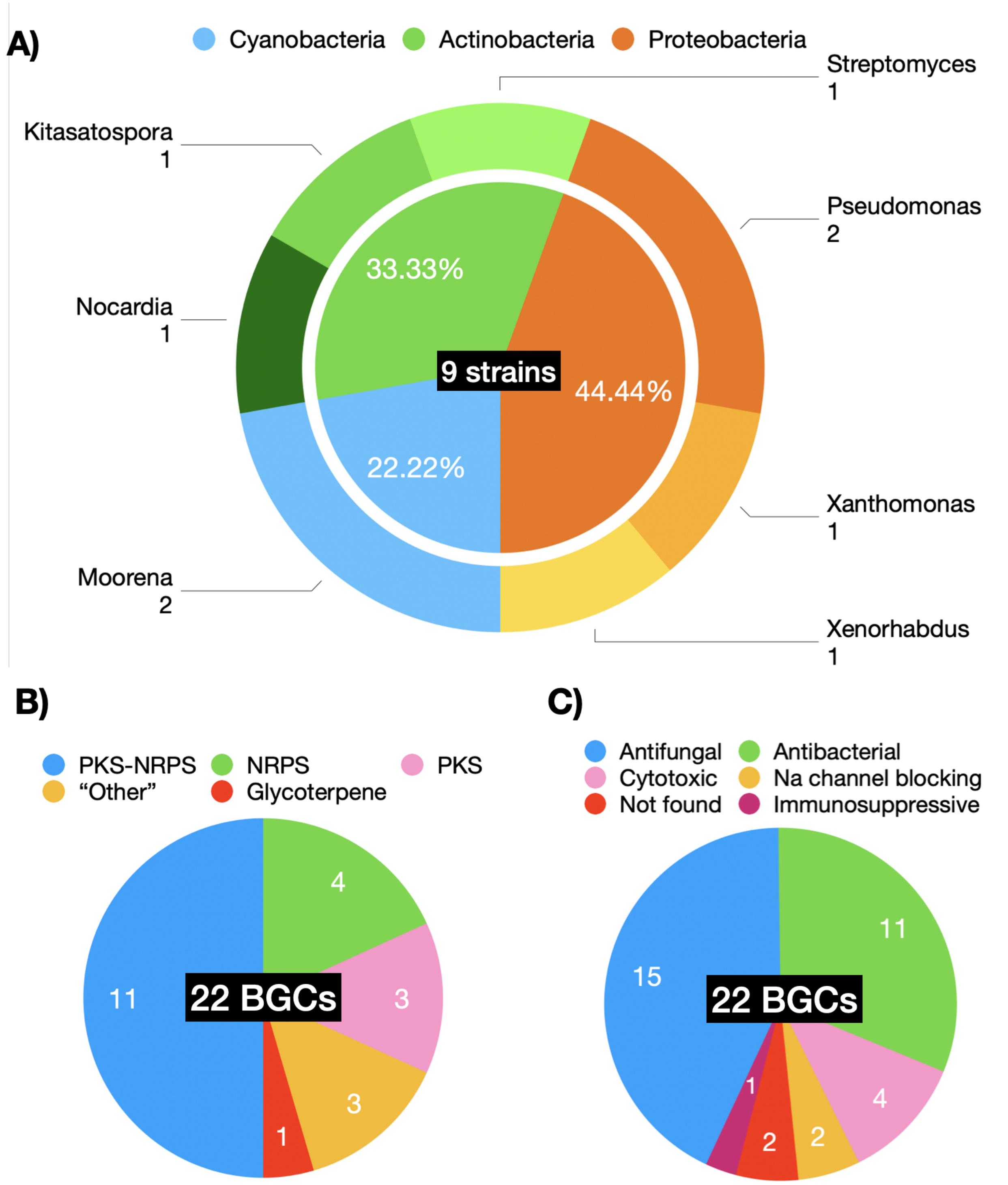

Fig. S9. Diversity of 9 different bacteria (in A) from the PoDP database used for benchmarking NPOmix, the biosynthetic classes (in B), and the reported bioactivity (in C) found in the metabolites connected to their BGCs used in the validation dataset. Na = sodium; PKS = polyketide synthase; NRPS = nonribosomal peptide-synthetase.

Table S1. The number of genomes, metabolomes, total BGCs, total GCFs, MIBiG BGCs networked, GNPS metabolites dereplicated, metabolites with MIBiG BGC (and the subset correctly linked by NPOmix), and metabolites with MIBiG BGC post-filtering with 0.7 Jaccard similarity (and the subset correctly liked by NPOmix). Pal* = to palmyramide A, which the BGC is not on MIBiG yet.

Type of feature in our dataset	
Number of networked genomes	1,040
Number of metabolomes	3,248
Number of networked BGCs	3,331
Number of GCFs	997
Number of BGCs in dereplicated GCFs	260
MIBiG BGCs networked	178
GNPS metabolites dereplicated (most of them are reported at PoDP)	50
Metabolites dereplicated with MIBiG BGC	22+Pal*
Metabolites dereplicated with MIBiG BGC and correctly linked by NPOmix	17+Pal*
Metabolites dereplicated with MIBiG BGC post-filtering	14
Metabolites dereplicated with MIBiG BGC post-filtering and correctly linked by NPOmix	13

Table S2. Top-*n* precision scores (how often the correct GCF label was found among the top *n* labels classified by the KNN approach) for around 14 references GNPS MS/MS spectra connected to a BGC found in the paired 1,040 (meta)genomes-MS/MS downloaded from the GNPS, MIBiG and PoDP databases (these links passed the co-occurrence threshold). These known links were obtained from the NPLinker dataset, GNPS, and PoDP databases. For this analysis, we only used similarity and biosynthetic classes as features because they are easier to annotate even from cryptic MS/MS spectra. Randomness is observed by shuffling the testing columns, experimental MS/MS names, and counting how many correct links are present between the top-*n* GCF candidates. Based on this, we believe the best performance is *n* = 3 (and it is the same value for k) for the examined dataset.

	Top-1	Top-3	Top-5	Top-10	Top-50	Top-100
Data	81.8%	92.9%	92.9%	92.9%	92.9%	92.9%
Random	0%	0%	0%	0%	0%	60%

Table S3. Multi-omics tools published to date, the type of links they can generate, and their websites for running samples or downloading their command-line version. Of note, only NPOmix, NPLinker, and GNP ("service unavailable") are systematic (work for many classes of natural products). MetaMiner, and DeepRiPP are specific for ribosomally synthesized and post-translationally modified peptides (RiPPs); nonribosomal peptides (NRPs) can be processed by NRPquest (apparently discontinued), NRPminer and Nerpa. Nerpa can also process hybrid polyketides-nonribosomal peptides and GARLIC can process NRPs, polyketides, and hybrids.

Tool	Type of links	Website for running samples	Command line version
NPOmix (this study)	BGC-MS/MS	https://www.tfleao.com/npomix1	https://github.com/tiagolbiotech/NPOmix_python
NPLinker (7)	BGC-MS/MS<br>BGC-SMILES	Not available	https://github.com/sdrogers/nplinker
GNP (22)	BGC-MS/MS	https://magarveylab.ca/gnp<br>("Service unavailable")	Not available
DeepRiPP (19)	BGC-MS/MS<br>BGC-SMILES	http://deepripp.magarveylab.ca	Not fully available<br>(only NLPPrecursor module)
MetaMiner (18)	BGC-MS/MS	https://gnps.ucsd.edu/ProteoSAFe/index.jsp?params=%7B%22workflow%22:%22RIPPQUEST%22%7D	https://github.com/ablab/npdtools/blob/master/docs/MetaMiner.md
NRPquest (20)	BGC-MS/MS	http://mohimanilab.cbd.cmu.edu/software/	Not available
NRPminer (21)	BGC-MS/MS	https://metabologenomic.cbd.cs.cmu.edu/#/login	https://github.com/mohimanilab/NRPminer (read me and test data only)
Nerpa (23)	BGC-SMILES	Not available	https://github.com/ablab/nerpa
GARLIC (24)	BGC-SMILES	Not available	https://github.com/magarveylab/garlic-release

References

1. T. Pluskal, S. Castillo, A. Villar-Briones, M. Orešič, MZmine 2: Modular framework for processing, visualizing, and analyzing mass spectrometry-based molecular profile data. *BMC Bioinformatics.* **11**, 395 (2010).
2. H. Mohimani, *et al.*, Dereplication of microbial metabolites through database search of mass spectra. *Nat. Commun.* **9**, 4035 (2018).
3. E. P. Balskus, C. T. Walsh, The Genetic and Molecular Basis for Sunscreen Biosynthesis in Cyanobacteria. *Science.* **329**, 1653–1656 (2010).
4. S. A. Kautsar, J. J. J. Van Der Hooft, D. De Ridder, M. H. Medema, BiG-SLiCE: A highly scalable tool maps the diversity of 1.2 million biosynthetic gene clusters. *Gigascience* **45**, W55–W63 (2021).
5. F. Huber, L. Ridder, S. Rogers, J. J. J. van der Hooft, Spec2Vec: Improved mass spectral similarity scoring through learning of structural relationships. *PLoS Comput. Biol.* **17**, e1008724 (2020).
6. F. Huber, S. van der Burg, J. J. J. van der Hooft, L. Ridder, MS2DeepScore - a novel deep learning similarity measure for mass fragmentation spectrum comparisons. *bioRxiv* (2021).
7. G. Hjörleifsson Eldjárn, *et al.*, Ranking microbial metabolomic and genomic links in the NPLinker framework using complementary scoring functions. *PLOS Comput. Biol.* **17**, e1008920 (2021).
8. K. Dührkop, *et al.*, SIRIUS 4: a rapid tool for turning tandem mass spectra into metabolite structure information. *Nat. Methods.* **16**, 299–302 (2019).
9. J. J. J. Van Der Hooft, *et al.*, Unsupervised Discovery and Comparison of Structural Families Across Multiple Samples in Untargeted Metabolomics. *Anal. Chem.* **89**, 7569–7577 (2017).
10. M. Ernst, *et al.*, Molnetenhancer: Enhanced molecular networks by integrating metabolome mining and annotation tools. *Metabolites.* **9**, 144 (2019).
11. K. Dührkop, *et al.*, Systematic classification of unknown metabolites using high-resolution fragmentation mass spectra. *Nat. Biotechnol.* **39**, 462–471 (2020).
12. A. S. Walker, J. Clardy, A Machine Learning Bioinformatics Method to Predict Biological Activity from Biosynthetic Gene Clusters. *J. Chem. Inf. Model.* **61**, 2560-2571 (2021).
13. A. K. Jarmusch, *et al.*, A Universal Language for Finding Mass Spectrometry Data Patterns. *bioRxiv* (2022).
14. J. J. R. Louwen, M. H. Medema, J. J. J. Van Der Hooft. Enhanced correlation-based linking of biosynthetic gene clusters to their metabolic products through chemical class matching. *Research Square* (2022).
15. A. Bankevich, *et al.*, SPAdes: A New Genome Assembly Algorithm and Its Applications to Single-Cell Sequencing. *J. Comput. Biol.* **19**, 455–477 (2012).
16. K. Blin, *et al.*, AntiSMASH 5.0: Updates to the secondary metabolite genome mining pipeline. *Nucleic Acids Res.* **47**(W1):W81-W87 (2019).
17. J. C. Navarro-muñoz, *et al.*, A computational framework to explore large-scale biosynthetic diversity. *Nat. Chem. Biol.* **47**, W81–W87 (2019).

18. L. Cao, *et al.*, MetaMiner: A Scalable Peptidogenomics Approach for Discovery of Ribosomal Peptide Natural Products with Blind Modifications from Microbial Communities. *Cell Syst.* **9**, 600-608.e4 (2019).
19. N. J. Merwin, *et al.*, DeepRiPP integrates multiomics data to automate discovery of novel ribosomally synthesized natural products. *Proc. Natl. Acad. Sci. U. S. A.* **117**, 371–380 (2020).
20. H. Mohimani, *et al.*, NRPquest: Coupling mass spectrometry and genome mining for nonribosomal peptide discovery. *J. Nat. Prod.* **77**, 1902–1909 (2014).
21. B. Behsaz, *et al.*, Integrating genomics and metabolomics for scalable non-ribosomal peptide discovery. *Nat. Commun.* **12**, 3225 (2021).
22. M. Wang, *et al.*, Sharing and community curation of mass spectrometry data with Global Natural Products Social Molecular Networking. *Nat. Biotechnol.* **34**, 828–837 (2016).
23. O. Kunyavskaya, *et al.*, Nerpa : A Tool for Discovering Biosynthetic Gene Clusters of Bacterial Nonribosomal Peptides. *Metabolites*. **11**(10), 693 (2021).
24. C. A. Dejong, *et al.*, Polyketide and nonribosomal peptide retro-biosynthesis and global gene cluster matching. *Nat. Chem. Biol.* **12**, 1007–1014 (2016).
